# Pharmacological treatment strategies for antipsychotic-induced hyperprolactinemia: a systematic review and network meta-analysis

**DOI:** 10.1038/s41398-022-02027-4

**Published:** 2022-07-05

**Authors:** Zhe Lu, Yaoyao Sun, Yuyanan Zhang, Yu Chen, Liangkun Guo, Yundan Liao, Zhewei Kang, Xiaoyang Feng, Weihua Yue

**Affiliations:** 1 Institute of Mental Health, Peking University Sixth Hospital, Beijing, 100191 China; 2 National Clinical Research Center for Mental Disorders, (Peking University Sixth Hospital), Beijing, 100191 China; 3 NHC Key Laboratory of Mental Health, Peking University, Beijing, 100191 China; 4 PKU-IDG/McGovern Institute for Brain Research, Peking University, Beijing, 100871 China; 5 Chinese Institute for Brain Research, Beijing, 102206 China

**Keywords:** Schizophrenia, Clinical pharmacology

## Abstract

Antipsychotic-induced hyperprolactinemia (AP-induced HPRL) occurs overall in up to 70% of patients with schizophrenia, which is associated with hypogonadism and sexual dysfunction. We summarized the latest evidence for the benefits of prolactin-lowering drugs. We performed network meta-analyses to summarize the evidence and applied Grading of Recommendations Assessment, Development, and Evaluation frameworks (GRADE) to rate the certainty of evidence, categorize interventions, and present the findings. The search identified 3,022 citations, 31 studies of which with 1999 participants were included in network meta-analysis. All options were not significantly better than placebo among patients with prolactin (PRL) less than 50 ng/ml. However, adjunctive aripiprazole (ARI) (5 mg: MD = −64.26, 95% CI = −87.00 to −41.37; 10 mg: MD = −59.81, 95% CI = −90.10 to −29.76; more than 10 mg: MD = −68.01, 95% CI = −97.12 to −39.72), switching to ARI in titration (MD = −74.80, 95% CI = −134.22 to −15.99) and adjunctive vitamin B6 (MD = −91.84, 95% CI = −165.31 to −17.74) were associated with significant decrease in AP-induced PRL among patients with PRL more than 50 ng/ml with moderated (adjunctive vitamin B6) to high (adjunctive ARI) certainty of evidence. Pharmacological treatment strategies for AP-induced HPRL depends on initial PRL level. No effective strategy was found for patients with AP-induced HPRL less than 50 ng/ml, while adjunctive ARI, switching to ARI in titration and adjunctive high-dose vitamin B6 showed better PRL decrease effect on AP-induced HPRL more than 50 ng/ml.

## Introduction

Patients with psychosis like schizophrenia spectrum disorder and bipolar disorder could benefit from the antipsychotics (APs) [1, 2], but also suffer from side-effects of APs which prolongs the optimum treatment time and influences the prognosis.

Hyperprolactinemia (HPRL) is a common side-effect of APs, relating to the blocking of dopamine receptors in in the tuberoinfundibular dopaminergic pathway, and occurs in around 70% patients receiving APs [3].

The mostly studied consequences of HPRL are amenorrhea, galactorrhea, sexual impairment and infertility. Most clinical guidelines addressing recommended only symptomatic AP-induced HPRL need to be treated [4], but the non-symptomatic HPRL also attracts a clinical attention because of its long-term outcomes. Prolactin, a pleiotropic hormone which is secreted into circulation and acts in a wide range of tissues, involves in a wide range of physiological function such as the immune function, reproductive function and metabolic function [5]. Pathological HPRL could lead to weight gain, increased fat mass, leptin insensitivity, insulin resistance, osteoporosis and even breast cancer [6–8], which impairs the physical health of patients accepted antipsychotics treatment. Currently, clinicians often focus on the HPRL-induced short-term or directly consequences like sexual impairment amenorrhea, the long-term outcomes of HPRL are often overlooked.

Several pharmacological strategies that can improve the AP-induced HPRL, such as adding adjunctive aripiprazole (ARI) [9–11], switching to another antipsychotic (e.g., ARI, quetiapine, olanzapine, clozapine, blonanserin, and brexpiprazole) [12–20], adding dopamine agonists (DA, e.g., cabergoline, bromocriptine) [21–23], adding metformin (MET) [24], or adding the Peony-Glycyrrhiza decoction (PGD) and other traditional herb treatments [25–29]. One recent study also suggested a potential option for adjunctive high-dose vitamin B6 [30]. The most important issue associated with those strategies is the risk of worsening psychopathology of psychosis, which is particularly high in the strategies of switching to another antipsychotic or adding dopamine agonist [31]. Some previous meta-analyses have addressed the role of ARI, PGD and MET in lowering prolactin (PRL) concentration [32–38], and a recent network meta-analysis (NMA) also compared the efficacy of ARI, PGD and MET on reducing PRL which suggested that adjunctive aripiprazole (<5 mg/day) was the most effective one [39].

When multiple studies are conducted on the same research question, even if the same protocol is used, the results obtained in different medical settings are not the same. Therefore, clinical studies need to be repeated. Meta-analysis of all evidence is crucial for decision-making. Meta-analysis can ensure the reproducibility of research results by using standardized operating procedures, systematically retrieving relevant research evidence for methodological evaluation, and comprehensively analyzing the results of a certain research question. Randomized controlled trials (RCTs) and their meta-analyses provide the highest level of evidence from epidemiological studies and are ideal for testing scientific hypotheses. However, RCTs are often expensive, time-consuming, and in some cases even unethical or unfeasible. Due to various limitations of realistic conditions, RCTs are often difficult to carry out or to meet the current demand for evidence. Single-arm trials are common in medical research, and single-arm meta-analysis will conduct quantitative comprehensive analysis of single-arm trials with the same purpose. A network meta-analysis (NMA) is a method of assessing the effects of multiple interventions that leverages all direct and indirect evidence to provide a more precise estimate of the relative relationship between interventions than a single direct or indirect estimate. In addition, even if some interventions have never been compared in RCTs, NMA can use indirect evidence to estimate the relative effects of these interventions. The Bayesian hierarchical model is a statistical model with a structured hierarchy, based on interchangeability. The core idea is to add random effect parameters to the model to reflect the correlation of data within a group and the heterogeneity between data in different groups. The Bayesian Hierarchical NMA model increases the precision of parameter estimates and preserves the interpretability of the intervention.

To our knowledge, there is no systematic reviews or meta-analysis that examine all strategies for lowering prolactin levels have been conducted yet. The aim of our study is to perform NMA [40] to compare the efficacy of all the above strategies in reducing AP-induced HPRL, and to test the results according different initial PRL levels, so that to provide a reasonable treatment suggestion for AP-induced HPRL.

## Methods

### Search strategy and selection criteria

We did a systematic review and network meta-analysis according to Preferred Reporting Items for Systematic Reviews and Meta-Analyses (PRISMA) guidelines. The protocol was registered in PROSPERO (CRD42022296815). Four electronic bibliographic databases were searched: PubMed, Web of Science, Embase and The Cochrane Library.

The search consisted of the following terms as Medical Subject Headings (MSH) and keywords appropriate to each database. The following search strategy was used: (schizo* OR psycho*) AND (switch* OR aripiprazole OR (bromocriptine OR cabergoline OR “dopamine agonist”) OR metformin OR (“peony-glycyrrhiza decoction” OR PGD OR herb*)) AND (prolactin OR PRL OR hyperprolactinemia).

### Eligibility criteria

Language was restricted to those articles written in English or Chinese. Abstract of Chinese study must be searched in the above sources, and the abstract must be in English. Studies published since inception to November 1st, 2021 were considered for inclusion.

Two authors (ZL and YS) completed the screening and recording independently, and they will not interfere with each other’s decisions. When there are different opinions between the two authors, the third author (YZ) clarified and made final decision.

According to PICOS acronym, the selection criteria were included as follows: Participants (*P*): we included studies in adults with schizophrenia (as diagnosed using any recognized diagnostic criteria) who were treated with antipsychotics and experienced hyperprolactinemia induced by antipsychotics. We excluded studies in subjects with significant medical illnesses (such as liver or renal dysfunction, cardiovascular disease, organic brain disorder), pregnancy or lactation, the psychiatric diagnosis other than schizophrenia and a current history of substance use disorder; Studies in teenagers (under 18 years old) and elder people (more than 65 years) were also excluded; Interventions (*I*): (1) previous antipsychotics plus adjunctive medication which could reduce the prolactin level; (2) switch previous antipsychotics to another antipsychotic; Comparators (*C*): previous antipsychotics plus placebo or antipsychotic monotherapy (studies without controls were analyzed in single-arm meta-analysis); Outcomes (*O*): the mean change of PRL levels (ng/ml) after treatment (with PRL level at baseline and endpoint or the change of PRL levels); Study design (*S*): single-arm study and case-control studies were applied for single-arm meta-analysis. Placebo-controlled and head-to-head randomized controlled trials (RCTs) that compared different strategies were applied for network meta-analysis. The mean baseline prolactin levels must more than 25 ng/ml (HPRL was defined as prolactin levels more than 25 ng/ml). Studies with small sample size (*n* < 5) were excluded.

### Data collection process

Two authors (ZL and YS) completed the screening and recording independently, and they could not interfere with each other’s decisions. When there are different opinions between the two authors, the third author (YZ) clarified and made final decision.

### Data abstraction and synthesis

The baseline PRL level and endpoint PRL level were extracted (must include the mean and standard deviation), and the change of prolactin between baseline and endpoint was also extracted, if any.

For studies with multiple treatment arms of the same type of interventional drug, the mean/SDs were combined following methods described in the Cochrane Handbook (https://training.cochrane.org/handbook/current) and elsewhere.

### Quality assessment

For single-arm and case-control studies, we applied the Methodological Index for Non-Randomized Studies (MINORS) score to assess the risk of bias [41]. For RCT studies, we applied the Cochrane Risk of Bias tool 2 (RoB 2) to assess the risk of bias [42]. For the outcomes of NMA, we applied the Grading of Recommendations, Assessment, Development, and Evaluation (GRADE) to evaluate the level of evidence, which provided the framework for rating the certainty of the evidence of each paired comparison as high, moderate, low, or very low [43].

Discrepancies were resolved through discussion, and if needed, a third author. The risk of bias was marked in both articles and survey results.

### Data analysis

All the statistical actions were conducted on the R software, based on the *meta* [44] (applied for single-arm meta-analysis and RCT meta-analysis on each strategy which includes more than three studies)*, gemtc* and *rjags* (applied for network meta-analysis) package [45].

We modeled the mean changes of prolactin levels with standard deviations and reported posterior mean difference (MD) with 95% confidence intervals (CIs). The concentration unit of prolactin was unified into nanogram per milliliter using a relevant conversion formula.

For single-arm meta-analysis (evaluate the effect after treatment) and RCT meta-analysis (evaluate the effect between strategy and placebo) on each strategy, use of the random or fixed effects model and the heterogeneity of meta-analysis was determined by *I*^*2*^ (50 was set as threshold). Meta-regression (for categorical variables, in the study, we included dosage of aripiprazole [5 mg or more than 5 mg per day] and baseline prolactin level [more than 100 ng/ml or not]; for continuous variables, we included the age, duration of trail and precent of male subjects), and sensitivity analyses (*metainf* code: exclude each included study individually) were preformed to examine the sources of heterogeneity. Subgroup analyses were conducted based on the result of meta-regression analysis. Funnel plot and Egger’s test were used to assess the publication bias.

To further test the comparative effectiveness among different treatment strategies, network meta-analysis with random-effects model was conducted. We assessed the Heterogeneity by *I*^*2*^. Node-split method was used to calculate the inconsistency between direct and indirect evidence. We compared the efficacy of different strategies using the surface under the cumulative ranking curve (SUCRA). Subgroup analysis (we included dosage of aripiprazole (5 mg, 10 mg, more than 5 mg per day) or different switching strategies (titration of aripiprazole and tardation of previous antipsychotics reduction [switch_ari_ti_ta], fixed dosage of aripiprazole and tardation of previous antipsychotics reduction [switch_ari_fixed_ta], fixed dosage of aripiprazole and reducing previous antipsychotics immediately [switch_ari_fixed_im]), and baseline prolactin level (<50 ng/ml, 50–100 ng/ml and >100 ng/ml) were preformed to examine the sources of heterogeneity.

All statistical differences were considered significant when the *P* < 0.05.

## Results

### Study selection and characteristics

**Fig. 1:**
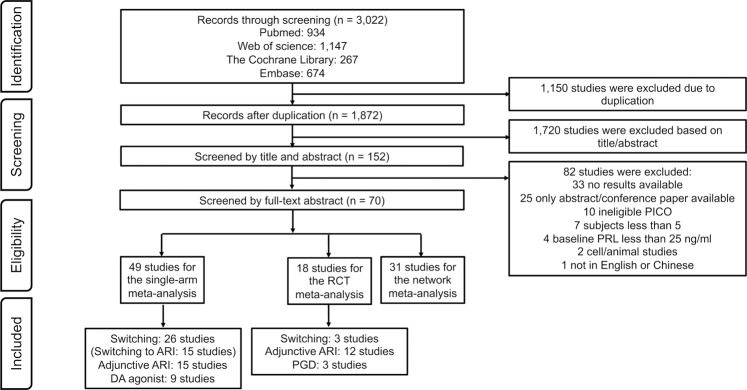
Flow chart of included studies. RCT randomized controlled trials, ARI aripiprazole, DA dopamine agonist, PGD Peony-Glycyrrhiza decoction.

The search identified 3022 citations, including 1872 unique reports, and 152 studies were retrieved after the screening by title and abstract, finally 70 full-text articles were included in the analysis (Table 1). Then the 49 studies were included for single-arm meta-analysis (26 studies with 1273 participants for switching strategy, 15 of which with 1015 participants were for switching to ARI strategy; 15 studies with 716 participants for adjunctive ARI strategy; 9 studies with 189 participants for adjunctive dopamine agonists), 18 studies were included for RCT meta-analysis (3 studies with 63 participants for switching strategy, 12 studies with 542 participants for adjunctive ARI strategy, 3 studies with 130 participants for adjunctive PGD strategy) and 31 studies with 1999 participants were included for network meta-analysis. Figure 1 shows the flow chart of the study selection.

**Table 1:** List of included studies.

First author (Publication Year)	Blind assessment	Sample size	Age (years) Mean	Male (%)	Trial duration	Diagnostic tools	Primary APs	Treatment	References
**Non-RCTs**									
Chen CY (2011)	Open-label	9	48.33	100	16 weeks	DSM-IV	RIS	Switching to ARI	[54]
Lu ML (2008)	Open-label	20	31.70	0	8 weeks	DSM-IV	RIS/SUL	Switching to ARI	[55]
Lee BH (2006)	Open-label	7	35	0	8 weeks	DSM-IV	RIS/AMI	Switching to ARI	[56]
Kinon BJ (2006)	Open-label	54	39.25	48.14	16 weeks	DSM-IV	Various	Switching to OLA	[18]
Nakajima M (2005)	Open-label	25	52.16	0	8 weeks	DSM-IV	Various	Switching to QUE	[57]
Takahashi H (2003)	Open-label	16	25.69	0	16 weeks	DSM-IV	RIS/HAL	Switching to QUE	[19]
Kawabe (2013)	Open-label	10	53.9	50	12 weeks	DSM-IV	Various	Switching to BLO	[58]
Hatzimanolis J (1998)	Open-label	17	33.3	NR	6 weeks	DSM-III	FGAs	Switching to CLO	[59]
Markianos M (1999)	Open-label	31	30.4	NR	6 weeks	DSM-III	FGAs	Switching to CLO	[20]
Kim KS (2002)	Open-label	20	34.4	0	8 weeks	DSM-IV	RIS	Switching to OLA	[60]
Takeuchi H (2010)	Open-label	32	54.6	56.3	56 weeks	DSM-IV	Various	Switching to ARI	[61]
Woo YS (2016)	Open-label	77	36.2	37.7	24 weeks	DSM-IV	Various	Switching to ARI	[62]
Kelly DL (2021)	Open-label	50	40.4	74	6 months	NR	PAL(LAI)	Switching to ARI(LAI)	[63]
Woo YS (2019)	Open-label	33	NR	NR	12 weeks	DSM-IV	Various	Switching to BLO	[13]
Ichinose M (2021)	Open-label	27	57.6	59.26	8 weeks	DSM-5	Various	Switching to BRE	[12]
Kinon BJ (2000)	Open-label	45	NR	NR	3 weeks	NR	RIS	Switching to OLA	[64]
Montejo AL (2009)	Open-label	20	38.4	65	6 months	NR	Various	Switching to QUE	[65]
Jen YW (2020)	Open-label	63	38.7	41.27	8 weeks	DSM-IV	Various	Switching to ARI	[66]
Takeuchi H (2008)	Open-label	53	53.74	56.6	14 weeks	DSM-IV	Various	Switching to ARI	[16]
Hashimoto N (2015)	Open-label	22	52.1	45.45	12 months	DSM-IV	Various	Switching to ARI	[14]
Kim SW (2009)	Open-label	61	30.8	44.3	26 weeks	DSM-IV	Various	Switching to ARI	[67]
Nishimoto M (2012)	Open-label	7	NR	NR	NR	NR	NR	Switching to ARI	[68]
Fujioi J (2017)	Open-label	21	41.3	42.86	24 weeks	NR	Various	Adjunctive ARI	[69]
Ziadi Trives M (2013)	Open-label	13	41	12.5	3 months	NR	RIS(LAI)	Adjunctive ARI	[70]
Van Kooten M (2011)	Open-label	12	47.6	91.7	16 weeks	DSM-IV	RIS(LAI)	Adjunctive ARI	[71]
Yasui-Furukori (2010)	Open-label	17	44	0	8 weeks	DSM-IV	RIS	Adjunctive ARI	[72]
Chen CK (2010)	Open-label	26	37.38	50	8 weeks	DSM-IV	RIS/AMI/SUL	Adjunctive ARI	[73]
Chen JX (2009)	Open-label	19	NR	NR	8 weeks	DSM-IV	RIS	Adjunctive ARI	[74]
Arnaiz A (2021)	Open-label	74	44.47	72.97	1 month	DSM-IV	RIS/PAL	Adjunctive ARI	[75]
Raveendranthan D (2018)	Open-label	16	29.4	23.08	24 months	ICD-10	RIS/AMI/OLA	Adjunctive ARI	[9]
Jung DU (2011)	Open-label	24	NR	0	3 months	DSM-IV	RIS	Adjunctive ARI	[76]
Sajeev Kumar PB (2010)	Open-label	10	NR	NR	48 weeks	NR	Various	Adjunctive ARI	[77]
Kalkavoura CS (2013)	Open-label	80	43.6	56.25	6 months	DSM-IV	Various	Adjunctive DA	[22]
Coronas R (2012)	Open-label	6	31.1	33.33	12 months	DSM-IV	Various	Adjunctive DA	[78]
Cavallaro R (2004)	Open-label	19	33.7	31.58	6 months	DSM-IV	RIS	Adjunctive DA	[79]
Bliesener N (2004)	Open-label	5	NR	NR	NR	DSM-IV	AMI	Adjunctive DA	[80]
Hashimoto (2014)	Open-label	20	42.9	50	2–4 weeks	DSM-IV	RIS/PAL	Adjunctive DA	[21]
Siever LJ (1981)	Open-label	11	NR	12.5	2 weeks	NR	FGAs	Adjunctive DA	[23]
Cohn JB (1985)	Open-label	11	NR	44.44	6 weeks	NR	THI	Adjunctive DA	[81]
**RCTs**									
Lee BJ (2013)	double	29	50.78	72.41	24 weeks	DSM-IV	RIS	Switch_ARI_ti_ta/Placebo	[82]
Ryckmans V (2009)	Open-label	400	41.10	56	12 weeks	DSM-IV	RIS	Switch_ARI_ti_ta / Switch_ARI_fixed_ta	[15]
Byerly MJ (2008)	double	42	42.30	52.38	8 weeks	NR	RIS	Switching to QUE/Placebo	[17]
Byerly MJ (2009)	Open-label	105	40	72.38	8 weeks	DSM-IV	RIS	Switch_ARI_fixed_im/Switch_ARI_fixed_ta/Switch_ARI_ti_ta	[11]
Huang P (2011)	Open-label	67	23.73	0	3 months	CCMD-3	Various	Switch_ARI_ti_ta/PGD	[83]
Hwang TJ (2015)	Open-label	79	39.52	40.5	8 weeks	DSM-IV	Various	Switch_ARI_fixed_im/Switch_ARI_fixed_ta	[84]
Chen JX (2015)	double	120	33.57	47.5	8 weeks	DSM-IV	RIS	ARI_5/ARI_10/ARI_more_10/Placebo	[85]
Shim JC (2007)	double	54	39.39	40.74	8 weeks	DSM-IV	HAL	ARI_more_10/Placebo	[86]
Kelly DL (2018)	double	42	37.03	0	16 weeks	DSM-IV	Various	ARI_10/Placebo	[87]
Qiao Y (2016)	single	60	33.35	0	8 weeks	DSM-IV	RIS/PAL	ARI_5/Placebo	[10]
Zhao J (2015)	single	107	29.67	41.12	8 weeks	DSM-IV	RIS	ARI_10/Placebo	[88]
Xu LP (2006)	single	60	25	0	6 weeks	CCMD-3	RIS/SUL	ARI_5/Placebo	[89]
Ji JY (2008)	single	117	25	0	6 weeks	CCMD-3	RIS	ARI_5/Placebo	[90]
Chen HZ (2009)	double	65	30.5	100	8 weeks	CCMD-3	RIS	ARI_5/Placebo	[91]
Liu ZB (2011)	Open-label	142	38.75	61.25	26 weeks	CCMD-3	Various	ARI_5/Placebo	[92]
Chen JX (2014)	double	116	34.04	63.79	8 weeks	ICD-10	RIS	ARI_more_10/Placebo	[93]
Liang J (2014)	double	40	30.45	37.5	4 weeks	DSM-IV	PAL	ARI_10/Placebo	[94]
Wang HL (2014)	double	178	34.69	50	6 weeks	CCMD-3	Various	ARI_5/ARI_10	[95]
Xia SY (2014)	Open-label	67	32.02	0	6 months	NR	Various	ARI_5/PGD	[27]
Xu CX (2015)	Open-label	193	36.78	41.96	12 weeks	CCMD-3	RIS/AMI	ARI_5/ARI_10/ARI_more_10/Placebo	[96]
Chen HM (2016)	double	61	33.43	0	8 weeks	DSM-IV	RIS	ARI_5/ARI_10/ARI_more_10/Placebo	[97]
Zhang LG (2018)	double	58	35.13	100	8 weeks	DSM-IV	RIS	ARI_5/ARI_10/ARI_more_10/Placebo	[98]
Wu RR (2012)	double	84	26.4	0	6 months	DSM-IV	Various	MET/Placebo	[24]
Xia JX (2011)	Open-label	143	NR	60.14	6 months	CCMD-3	RIS	MET/Placebo	[99]
Yuan HN (2008)	single	20	30.45	0	12 weeks	ICD-10	RIS	DA/Placebo	[100]
Yu RL (2010)	Open-label	63	26.15	0	12 weeks	NR	Various	DA/PGD	[28]
Yang P (2017)	double	42	28.48	0	8 weeks	ICD-10	AMI	PGD/Placebo	[25]
Man SC (2016)	double	99	29.8	0	16 weeks	ICD-10	Various	PGD/Placebo	[26]
Gu P (2016)	Open-label	120	30.22	44.17	8 weeks	ICD-10	OLA	PGD/Placebo	[101]
Zhuo C (2021)	double	200	31.82	100	16 weeks	DSM-IV	Various	Vitamin B6/ARI_10	[30]
Yoon HW (2016)	Open-label	42	35.34	33.33	8 weeks	DSM-IV	Various	Switching to ARI /Adjunctive ARI	[102]

*ARI_5* *mg* adjunctive 5 mg aripiprazole, *ARI_10* *mg* adjunctive 10 mg aripiprazole, *ARI_more_10* *mg* adjunctive more than 10 mg aripiprazole, *DA* adjunctive dopamine agonist, *MET* adjunctive metformin, *PGD* adjunctive Peony-Glycyrrhiza decoction, *switch_ARI_fixed_im* switching to ARI with fixed dosage and reducing the previous antipsychotic immediately, *switch_ARI_fixed_ta* switching to ARI with fixed dosage and reducing the previous antipsychotic in tardation, *switch_ARI_ti_ta* switching to ARI in titration and reducing the previous antipsychotic in tardation, *switch_OLA* switching to olanzapine, *switch_QUE* switching to quetiapine, *VitB6* adjunctive high-dose vitamin B6, *RIS* risperidone, *SUL* sulpiride, *AIM* amisulpride, *HAL* haloperidol, *FGAs* first-generation antipsychotics, *PAL* paliperidone, *LAI* long-acting injection, *THI* thioridazine, *OLA* olanzapine, *DSM-IV* Diagnostic and Statistical Manual of Mental Disorders, fourth version, *ICD-10* International Statistical Classification of Diseases and Related Health Problems, 10th Revision, *CCMD-3* Chinese Classification of Mental Disorders, 3rd version, *NR* not report.

### Single-arm meta-analysis

#### Switching strategy

The result of switching strategy was significant. Switching to another antipsychotic could significantly reduce the prolactin levels (Supplementary Fig. 2, MD = − 42.55 ng/ml, 95% CI = −61.47 to −37.19 ng/ml). These studies were quite heterogeneous (*I*^*2*^ = 98%), so a random-effect model was used to generate the pooled estimates. Egger’s test results indicate there was no statistically significant level of publication bias (Supplementary Fig. 3A, *t* = 0.01, *df* = 24, *P* = 0.9932). Sensitivity analyses found that the random model was stable (Supplementary Fig. 3B). Test of heterogeneity also indicated the high heterogeneity (*Q* = 1,119.48, *df* = 25, *P* < 0.0001), so we applied the age, sex (male participants rate), baseline PRL level, medication type and trail duration to detect the contributions of heterogeneity, the result showed that the sex and baseline PRL level was significant.

#### Switching to ARI

Switching to ARI could significantly reduce the prolactin levels (Supplementary Fig. 4, MD = −55.79 ng/ml, 95% CI = −72.74 to −38.85 ng/ml). These studies were quite heterogeneous (*I*^*2*^ = 98%), so a random-effect model was used to generate the pooled estimates. Egger’s test results indicate there was no statistically significant level of publication bias (Supplementary Fig. 5A, *t* = −0.03, *df* = 13, *P* = 0.9760). Sensitivity analyses found that the random model was stable (Supplementary Fig. 5B). Test of heterogeneity also indicated the high heterogeneity (*Q* = 773.01, *df* = 16, *P* < 0.0001), so we applied the age, sex (male participants rate), baseline PRL level, dosage of ARI and trail duration to detect the contributions of heterogeneity, the result showed that baseline PRL level was significant.

#### Adjunctive ARI

Adjunctive ARI could significantly reduce the prolactin levels (Supplementary Fig. 6, MD = −46.31 ng/ml, 95% CI = −57.77 to −34.84 ng/ml). These studies were quite heterogeneous (*I*^*2*^ = 97%), so a random-effect model was used to generate the pooled estimates. Egger’s test results indicate a statistically significant level of publication bias (Supplementary Fig. 7A, *t* = −2.20, *df* = 14, *P* = 0.0454). Sensitivity analyses found that the random model was stable (Supplementary Fig. 7B). Test of heterogeneity also indicated the high heterogeneity (*Q* = 738.30, *df* = 21, *P* < 0.0001), so we applied the age, sex (male participants rate), baseline PRL level, dosage of ARI and trail duration to detect the contributions of heterogeneity, the result showed that baseline PRL level was significant.

#### Adjunctive dopamine agonist

Adjunctive dopamine agonist could significantly reduce the prolactin levels (Supplementary Fig. 8, MD = −40.29 ng/ml, 95% CI = −57.19 to −23.39 ng/ml). These studies were quite heterogeneous (*I*^*2*^ = 85%), so a random-effect model was used to generate the pooled estimates. Egger’s test results indicate there was no statistically significant level of publication bias (Supplementary Fig. 9A, *t* = −0.43, *df* = 7, *P* = 0.6795). Sensitivity analyses found that the random model was stable (Supplementary Fig. 9B). Test of heterogeneity also indicated the high heterogeneity (*Q* = 63.95, *df* = 9, *P* < 0.0001), so we applied the age, sex (male participants rate), baseline PRL level and trail duration to detect the contributions of heterogeneity, above factors were all not significant.

### RCT meta-analysis

#### Switching strategy

Only 3 studies were included in the analysis, and the result was not significant (*P* = 0.11), which might due to the high heterogeneity, their strategies were switching to ARI, OLA or QUE separately (Supplementary Fig. 10).

#### Adjunctive ARI

**Fig. 2:**
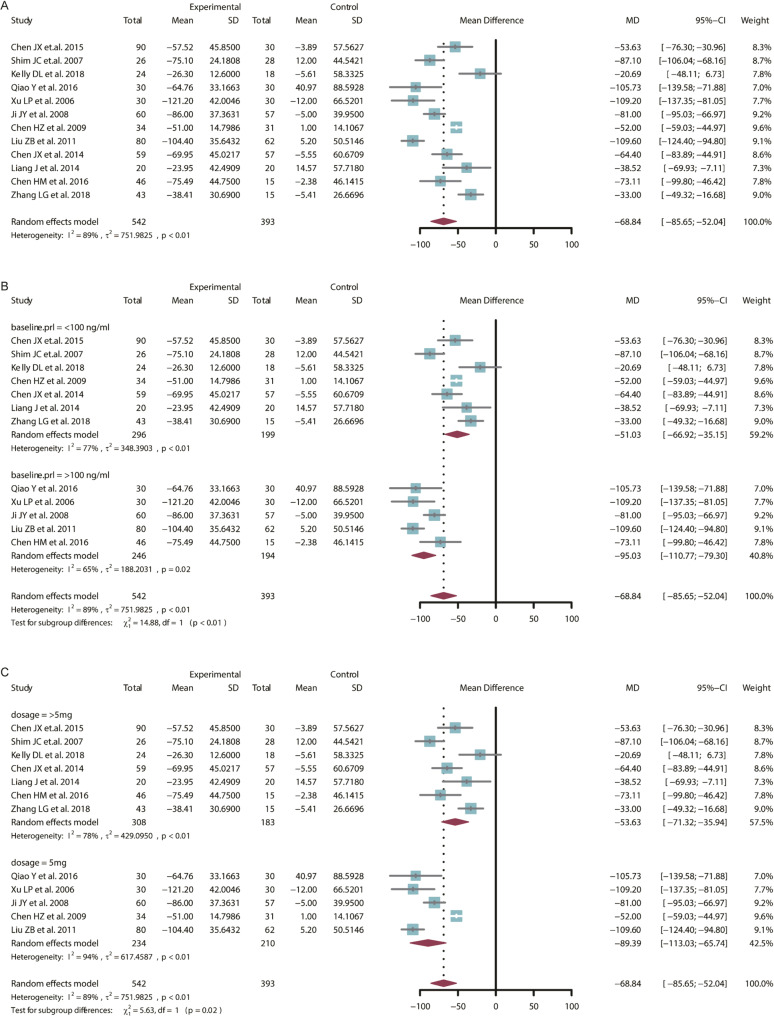
RCT meta-analysis of adjunctive aripiprazole. **A** Forest plot of RCT meta-analysis; **B** subgroup analysis based on baseline PRL level; **C** subgroup analysis based on ARI dosage. RCT randomized controlled trials, PRL prolactin, ARI aripiprazole, MD mean difference, CI confidence intervals, SD standard difference.

Compared to placebo, adjunctive ARI could significantly reduce the prolactin levels (Fig. 2A, MD = −68.84 ng/ml, 95% CI = −85.65 to −52.04 ng/ml). These studies were quite heterogeneous (*I*^*2*^ = 89%), so a random-effect model was used to generate the pooled estimates. Egger’s test results indicate there was no statistically significant level of publication bias (Supplementary Fig. 11A, *t* = −0.86, *df* = 10, *P* = 0.4094). Sensitivity analyses found that the random model was stable (Supplementary Fig. 11B). Test of heterogeneity also indicated the high heterogeneity (*Q* = 182.90, *df* = 14, *P* < 0.0001). According to the meta-regression analysis of the single-arm meta-analysis of adjunctive ARI, we conducted subgroup analysis to detect the heterogeneity. When we conducted the subgroup analysis stratified by the baseline PRL level (we set the 50 and 100 ng/ml as threshold, which were 2 times and 4 times of the normal limit; because there was no study with baseline PRL less than 50 ng/ml, we divided all the studies into two subgroup based on the 100 ng/ml), the PRL reduction after treatment in subgroup of lower baseline PRL (less than 100 ng/ml) was less than the subgroup of higher baseline PRL (more than 100 ng/ml) (Fig. 2B, C, MD: −51.03 v.s. −95.03 ng/ml). When we conducted the subgroup stratified by the dosage of ARI, the PRL reduction after treatment in subgroup of 5 mg/d was more than the subgroup of more than 5 mg/d (MD: −89.39 v.s. −53.63 ng/ml), it indicated that patients with antipsychotic-induced HPRL might be benefited from the low dosage of ARI more.

#### Adjunctive PGD

The effect of PGD on reducing PRL level was not significant (Supplementary Fig. 12A, MD = −11.76, 95%CI = −31.41 to 8.07). Egger’s test results indicate there was no statistically significant level of publication bias (Supplementary Fig. 12B, *t* = −0.43, *df* = 7, *P* = 0.6795). Sensitivity analyses found that the random model came to stable when we omitted the study of Man SC et al. (Supplementary Fig. 12C). Test of heterogeneity also indicated the high heterogeneity (*Q* = 19.68, *df* = 3, *P* = 0.0002).

#### Network meta-analysis

**Fig. 3:**
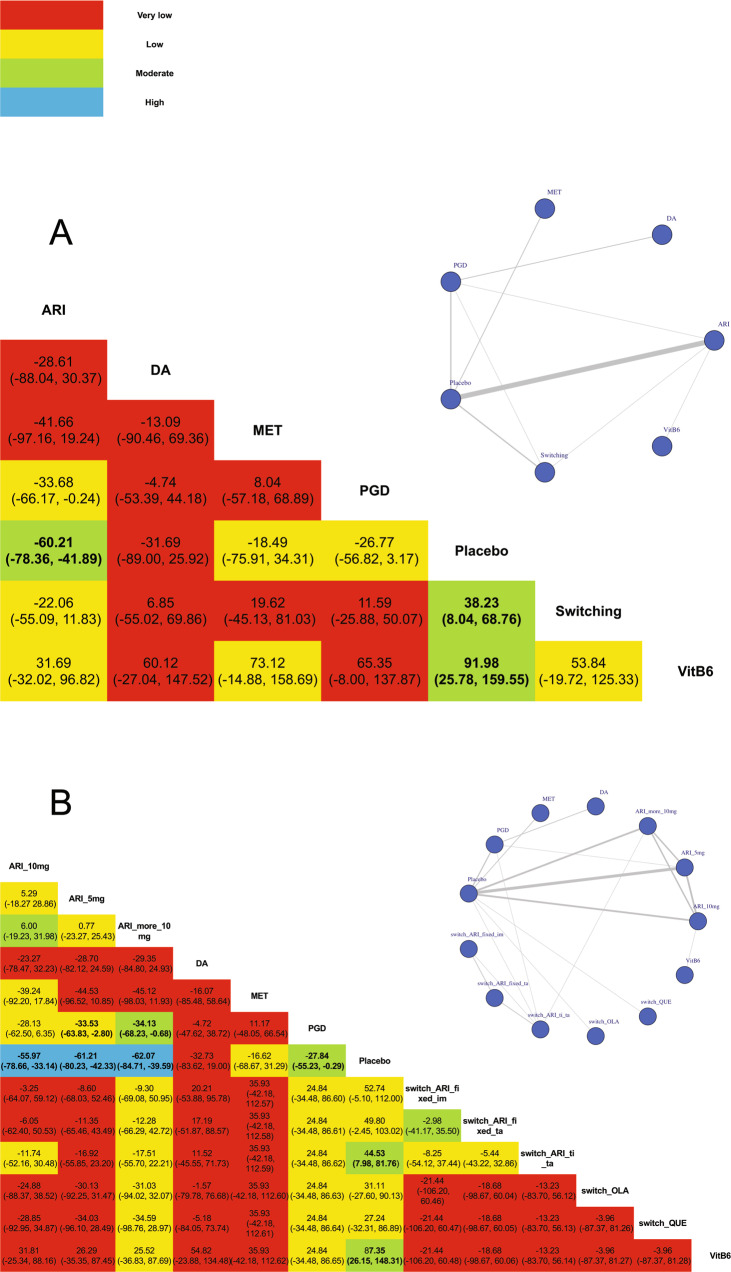
Network meta-analyses of all the strategies in treatment of patients with antipsychotic-induced hyperprolactinemia. **A** Network plot and league table of comparison of all the strategies in treatment of patients with antipsychotic-induced hyperprolactinemia; **B** network plot and league table of comparison of all re-divided strategies in treatment of patients with antipsychotic-induced hyperprolactinemia. ARI adjunctive aripiprazole, DA adjunctive dopamine agonist, MET adjunctive metformin, PGD adjunctive Peony-Glycyrrhiza decoction, Switching switch to another antipsychotic, VitB6 adjunctive high-dose vitamin B6. ARI_5 mg adjunctive 5 mg aripiprazole, ARI_10 mg adjunctive 10 mg aripiprazole, ARI_more_10 mg adjunctive more than 10 mg aripiprazole, switch_ARI_fixed_im switching to ARI with fixed dosage and reducing the previous antipsychotic immediately, switch_ARI_fixed_ta switching to ARI with fixed dosage and reducing the previous antipsychotic in tardation, switch_ARI_ti_ta switching to ARI in titration and reducing the previous antipsychotic in tardation, switch_OLA switching to olanzapine, switch_QUE switching to quetiapine. The color of each cell indicates the certainty of evidence according to the Grading of Recommendations Assessment, Development, and Evaluation. Red color refers to very low certainty of evidence, yellow color refers to low certainty of evidence, green color refers to moderate certainty of evidence, blue color refers to high certainty of evidence. The significant outcomes were shown in bold.

First of all, we conducted network meta-analysis on effect of different strategies (including adjunctive ARI, switching to another antipsychotic, adjunctive PGD, adjunctive MET, adjunctive DA, and adjunctive high-dose vitamin B6) on antipsychotic-induced HPRL, 26 studies with 1999 participants were included (5 studies was excluded because these head-to-head studies compared the different switching strategies or adding different dosage of ARI). When comparing to the placebo, adjunctive ARI (MD = −60.21, 95% CI = −78.36 to −41.89), switching to another antipsychotic (MD = −38.23, 95% CI = −68.76 to −8.04) and adjunction vitamin B6 (MD = −91.98, 95% CI = −159.55 to −25.78) showed the significant effect of decreasing PRL level. Furthermore, adjunctive ARI (MD = −33.68, 95% CI = −66.17 to −0.24) showed a more significant effect of decreasing PRL than adjunctive PGD. (Fig. 3A, B).

Treatment strategies on adding adjunctive ARI and switching to another antipsychotic were further divided according to dosage of ARI and switching antipsychotic medications. Three ARI subgroups (5 mg ARI, adjunctive 10 mg ARI, adjunctive more than 10 mg ARI) and 5 switching subgroups (witching to ARI with fixed dosage and reducing the previous antipsychotic immediately (switch_ARI_fixed_im), switching to ARI with fixed dosage and reducing the previous antipsychotic in tardation (switch_ARI_fixed_ta), switching to ARI in titration and reducing the previous antipsychotic in tardation (switch_ARI_ti_ta), switching to OLA, switching to QUE) were performed. Finally, 31 studies with 2954 participants were included. When comparing to the placebo, adjunctive ARI (5 mg: MD = −61.21, 95% CI = −80.23 to −42.33; 10 mg: MD = −55.97, 95% CI = −78.66 to −33.14; more than 10 mg: MD = −62.21, 95% CI = −80.23 to −42.33), adjunctive PGD (MD = −27.84, 95% CI = −55.23 to −0.29), switch_ARI_ti_ta (MD = −44.53, 95% CI = −81.76 to −7.98) and adjunctive vitamin B6 (MD = −87.35, 95% CI = −148.31 to −26.15) showed the significant effect of decreasing PRL level. Furthermore, adjunctive ARI 5 mg (MD = −33.53, 95% CI = −63.83 to −2.80) and more than 10 mg (MD = −34.13, 95% CI = −68.23 to −0.68) showed more significant effect of decreasing PRL than adjunctive PGD. (Fig. 3C, D).

#### Subgroup analysis

When it comes to the effect of baseline PRL on the NMA model, we conducted subgroup analysis based on different baseline PRL levels (including less than 50 ng/ml and more than 50 ng/ml; furthermore, we further analyzed the subgroup of more than 100 ng/ml).

**Fig. 4:**
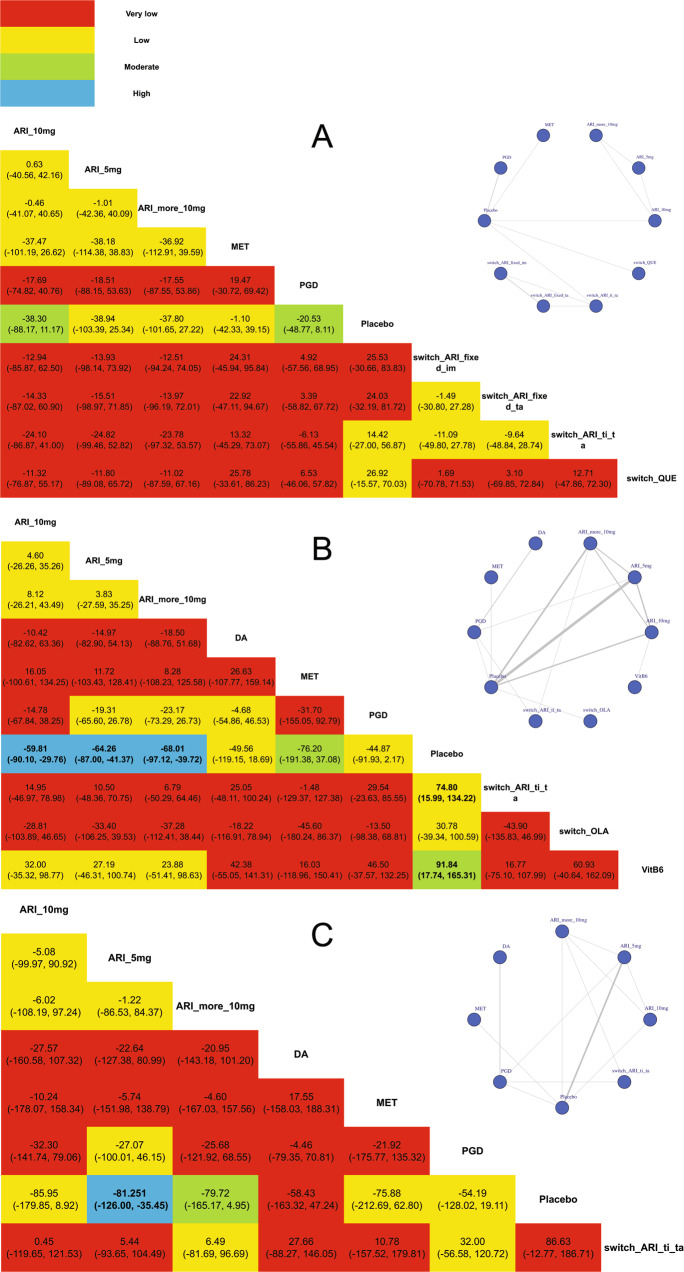
Subgroup analyses of network meta-analysis. **A** Network plot and league table of comparison of pharmacological treatment strategies in treatment of patients with antipsychotic-induced hyperprolactinemia less than 50 ng/ml; **B** network plot and league table of comparison of pharmacological treatment strategies in treatment of patients with antipsychotic-induced hyperprolactinemia more than 50 ng/ml; **C** network plot and league table of comparison of pharmacological treatment strategies in treatment of patients with antipsychotic-induced hyperprolactinemia more than 100 ng/ml. ARI_5 mg adjunctive 5 mg aripiprazole, ARI_10 mg adjunctive 10 mg aripiprazole, ARI_more_10 mg adjunctive more than 10 mg aripiprazole, MET adjunctive metformin, PGD adjunctive Peony-Glycyrrhiza decoction, switch_ARI_fixed_im switching to ARI with fixed dosage and reducing the previous antipsychotic immediately, switch_ARI_fixed_ta switching to ARI with fixed dosage and reducing the previous antipsychotic in tardation, switch_ARI_ti_ta switching to ARI in titration and reducing the previous antipsychotic in tardation, VitB6 adjunctive high-dose vitamin B6. The color of each cell indicates the certainty of evidence according to the Grading of Recommendations Assessment, Development, and Evaluation. Red color refers to very low certainty of evidence, yellow color refers to low certainty of evidence, green color refers to moderate certainty of evidence, blue color refers to high certainty of evidence. The significant outcomes were shown in bold.

In the subgroup of less than 50 ng/ml, 9 strategies were included (9 studies with 792 participants). There was no significant difference of PRL change after treatment between therapy and placebo. (Fig. 4A, B).

In the subgroup of more than 50 ng/ml, 9 strategies were included (21 studies with 1762 participants). When comparing to the placebo, adjunctive ARI (5 mg: MD = −64.26, 95% CI = −87.00 to −41.37; 10 mg: MD = −59.81, 95% CI = −90.10 to −29.76; more than 10 mg: MD = −68.01, 95% CI = −97.12 to −39.72), switch_ARI_ti_ta (MD = −74.80, 95% CI = −134.22 to −15.99) and vitamin B6 (MD = −91.84, 95% CI = −165.31 to −17.74) showed the significant effect of decreasing PRL level. (Fig. 4C, D).

In the subgroup of more than 100 ng/ml, 7 strategies were included (12 studies with 881 participants). When comparing to the placebo, only adjunctive 5 mg ARI (MD = −81.25, 95% CI = −126.00 to −35.45) showed the significant effect of decreasing PRL. (Fig. 4E, F).

#### Safety

Finally, we evaluated the incidence of side-effects of all strategies (including ARI_5 mg, ARI_10 mg, ARI_more_10 mg, MET, PGD, switch_OLA, and VitB6), 15 studies and 844 participants were included, the result showed that only ARI_more_10 mg (OR = 2.2, 95% CI = 1.2 to 4.3) were associated with higher incidence of side-effects compared to placebo.

## Discussion

To the best of our knowledge, this was the first NMA to comparing the efficacy of all strategies (6 strategies in total, including adjunctive ARI, switching to another antipsychotic, adjunctive PGD, adjunctive MET, adjunctive DA and adjunctive high-dose vitamin B6) for reducing AP-induced HPRL. Before we conducted the NMA, we firstly conducted the single-arm and RCT meta-analysis, the results showed that adjunctive ARI, switching to another antipsychotic, and adjunctive DA were associated with the significant decrease in AP-induced prolactin levels.

When we directly compared the six options, the NMA result showed that the adjunctive ARI, switching to another antipsychotic and adjunctive high-dose vitamin B6 was associated with the significant decrease in AP-induced prolactin levels compared to the placebo, and provided the moderated certainly evidence. Because there were some head-to-head RCTs which compared different dosage of ARI and switching to different antipsychotics, so we conducted the NMA included 12 options (adjunctive 5 mg ARI, adjunctive 10 mg ARI, adjunctive more than 10 mg ARI, switch_ARI_fixed_im, switch_ARI_fixed_ta, switch_ARI_ti_ta, switching to OLA, switching to QUE, adjunctive PGD, adjunctive MET, adjunctive dopamine agonist, and adjunctive vitamin B6), the result showed that the adjunctive ARI (in all subgroup), adjunctive PGD, switch_ARI_ti_ta and adjunctive vitamin B6 was associated with the significant decrease in AP-induced PRL compared to the placebo, and provided the high (adjunctive ARI) to moderated (adjuntive vitamin B6) certainly evidence; furthermore, the paired comparation indicated that the adjunctive ARI 5 mg was more efficacious than adjunctive PGD, while the certainly evidence was low. Baseline PRL was also a key-factor which influenced the change of PRL after treatment. We divided those studies into 2 subgroups based on the baseline PRL level (less than 50 ng/ml and more than 50 ng/ml), then we want to explore the best strategy for high PRL level (more than 100 ng/ml) and we set the studies whose baseline PRL more than 100 ng/ml as a subgroup. The result showed that all the options were not significant compared placebo for the patients with PRL level less than 50 ng/ml, which indicated that the intervention for patient with AP-induced HPRL less than 50 ng/ml might be not necessary; adjunctive ARI (all the subgroups: 5 mg, 10 mg and more than 10 mg), switch_ARI_ti_ta (low certainly evidence) and adjunctive high-dose vitamin B6 were associated with the significant decrease in AP-induced PRL compared to the placebo for the patients with PRL more than 50 ng/ml, and provided the high (adjunctive ARI) to moderated (adjunctive vitamin B6) certainly evidence; only the adjunctive 5 mg ARI was associated with the significant decrease in AP-induced PRL compared to the placebo for the patients with PRL more than 100 ng/ml.

The advantage of ARI, and PGD in reducing HPRL is consistent with previous researches [33, 35, 38]. The NMA of Zhang L et al. compared the efficacy among ARI, PGD and MET, it indicated that adjunctive ARI (<5 mg) was associated with the most significant reduction in prolactin levels compared to placebo; they also found that adjunctive PGD had the most significant effect in reducing risperidone-induced HPRL; furthermore, adjunctive aripiprazole (<5 mg) had the most significant effect in reducing amisulpride-induced HPRL, while the result was imprecise [39]. In our study, adjunctive ARI was also significantly associated with decrease in AP-induced HPRL, especially adjunctive ARI 5 mg in subgroup of baseline PRL more than 100 ng/ml, while this option was not significant in the subgroup of baseline PRL less than 50 ng/ml; adjunctive PGD was associated with AP-induced HPRL when we subdivided all the strategies. PGD is prepared from peony and glycyrrhiza in a certain proportion, which has been applied to improve HPRL in China and Japan. Its effect is associated with the modulation of dopamine D2 receptor. However, the preparation of PGD is various, and this strategy should be replicated in other countries. Adjunctive MET had no significant effect on reducing PRL compared to placebo, which was inconsistent with previous reviews [36, 37].

As for the switching strategy, there was no previous study, we did the single-arm meta-analysis firstly in this study, the result showed that the option was significant, but the result of RCT meta-analysis was not significant. The main reason might be switching to different antipsychotics. In the NMA, switching strategy was significant compared to placebo, especially for the switch_ARI_ti_ta option in the subgroup of PRL more than 50 ng/ml, while this option was not effective in the subgroup of PRL more than 100 ng/ml.

When it comes to the strategy of adjunctive DA, because the RCT number was too small, we just conducted the single-arm meta-analysis and it indicated this option was associated with decrease of AP-induced HPRL. However, the NMA result showed this strategy was not significantly associated with decrease of AP-induced HPRL.

Adjunctive high-dose vitamin B6, a recent novel attempt of old drug, was applied to treat the AP-induced HPRL, which showed a significant benefit for the participants. From 1970s, several case reports showed that high-dose vitamin B6 (from 200 to 1200 mg/day) could improve the galactorrhea-amenorrhea syndrome with or without hyperprolactinemia (including the drug-induced hyperprolactinemia) [46, 47], while there were some opposite results [48–50]. Due to the inconsistent curative effect and considering the adverse effect of high-dose vitamin B6, few studies were conducted to evaluate the potential efficacy. However, vitamin B6 is supposed that it works by promoting dopamine production and then activates dopamine receptors to reduce the secretion of pituitary prolactin [51], and the side-effects of high-dose vitamin B6 are fewer than expected [52], re-attempts of it are conducted recently and the results indicated that it could improve the AP-induced HPRL with few side-effects [30, 53]. In our study, the results of NMA showed that this option might be a very effective method for the patients with AP-induced HPRL and provide a moderate certainly evidence. However, there was only one study about it, this moderate evidence needed to be further researched in the future [30].

Because the strategies act in different mechanisms, the side effects of different strategies are various and affect a wide range of system including nervous system (insomnia, somnolence, agitation, anxiety/depression, psychosis, sedation, weakness, akathisia, tremor), metabolic system (liver dysfunction, elevated blood sugar), autonomic nervous system (nausea, salivate, constipation, dry mouth, rhinitis, diarrhea, stomachache) and cardiovascular system (tachycardia, electrocardiogram ST segment elevation). In order to evaluate the safety of each strategy, we extracted the total side-effect incidence rate and conducted the NMA. The result showed that ARI_more_10 mg were associated with higher incidence of side-effects compared to placebo. Combined the result of efficacy, low-dose ARI might be a better choice.

Strengths of our review included the most comprehensive synthesis of evidence to date on benefits of pharmacological therapies for adults with AP-induced HPRL, capturing all recent publications. And we conducted subgroup analysis based on the baseline PRL, which was more reasonable for clinical practice. We used state-of-the-art approaches to categorize and present the findings using GRADE frameworks. Limitations of our review included the absence of individual patient data pooling, which particularly reduced the precision of synthesis for subgroup effects. Studies varied in population characteristics and duration of follow-up. However, our meta-regression analyses showed no important differences in results across age and follow-up durations. In addition, some options like adjunctive vitamin B6 only included one publication, it might lack the representative.

In conclusion, patients with AP-induced HPRL could benefit from the strategies of adjunctive ARI, adjunctive Vitamin B6, adjunctive PGD and switching to ARI in titration. Patients with initial PRL less than 50 ng/ml might not need special intervention; adjunctive ARI, switch_ARI_ti_ta and adjunctive high-dose vitamin B6 proved to be the better PRL decrease effect for AP-induced HPRL more than 50 ng/ml; only adjunctive ARI 5 mg showed the significant effect of reduction PRL when the patients with AP-induced HPRL more than 100 ng/ml. Most comparative medium or high certainty evidence requires the confident application of these findings as clinical practice guidelines.

## Supplementary information

SUPPLEMENTAL MATERIAL

## Data Availability

The data and codes in this study are available from the corresponding author on reasonable request.
