# Supplementary material for: Pharmacological treatment strategies for antipsychotic-induced hyperprolactinemia: a systematic review and network meta-analysis
Source: Transl Psychiatry. 2022 Jul 5;12:267. doi: 10.1038/s41398-022-02027-4 (PMC9256633; doi:10.1038/s41398-022-02027-4)
Supplement: Supplementary file 1 — SUPPLEMENTAL MATERIAL [file 41398_2022_2027_MOESM1_ESM.pdf]

Supplementary Appendix

Supplementary Figures

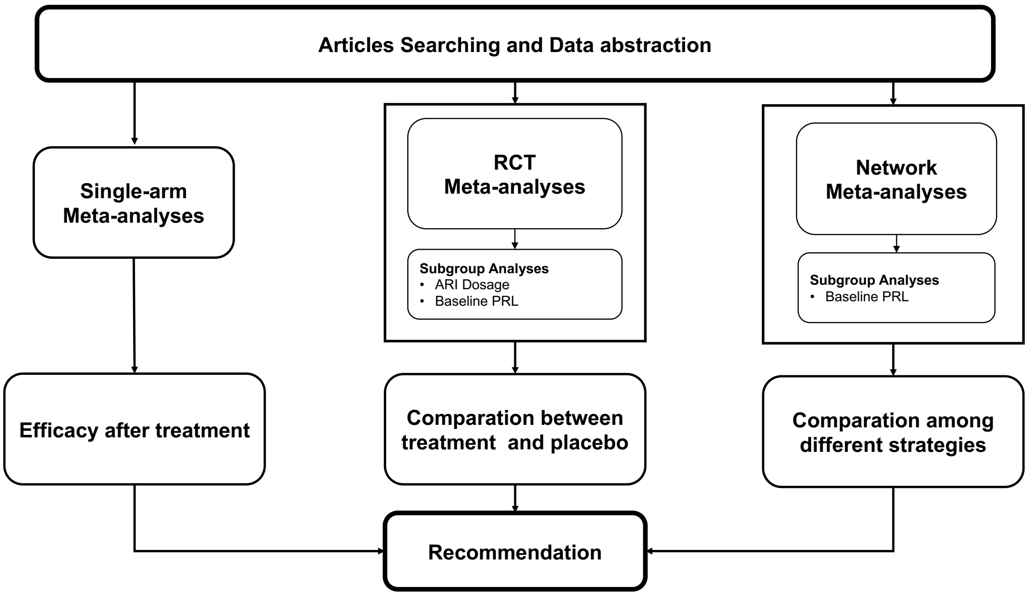

Supplementary Figure 1. Study framework.

Study	Total	Mean	SD	MRAW	95%-CI	Weight (common)	Weight (random)
Yoon HW et.al. 2016	18	-101.70	55.9365	-101.70	[-127.54; -75.86]	0.0%	3.5%
Chen CY et.al. 2011	9	-22.83	16.1763	-22.83	[-33.40; -12.26]	0.3%	4.1%
Lu ML et.al. 2008	20	-84.80	66.5086	-84.80	[-113.95; -55.65]	0.0%	3.4%
Lee BH et.al. 2006	7	-157.50	56.4680	-157.50	[-199.33; -115.67]	0.0%	2.8%
Nakajima M et.al. 2005	17	-40.40	28.2340	-40.40	[-53.82; -26.98]	0.2%	4.0%
Takahashi H et.al 2003	16	-84.70	10.2093	-84.70	[-89.70; -79.70]	1.2%	4.2%
Kawabe et al. 2013	10	-39.70	45.1479	-39.70	[-67.68; -11.72]	0.0%	3.4%
Hatzimanolis J et.al. 1998	17	-23.30	20.7478	-23.30	[-33.16; -13.44]	0.3%	4.1%
Markianos M et.al. 1999	31	-31.60	23.9940	-31.60	[-40.05; -23.15]	0.4%	4.1%
Kim KS et.al. 2002	20	-108.80	51.6385	-108.80	[-131.43; -86.17]	0.1%	3.7%
Ryckmans V et.al. 2009	400	-48.60	8.0852	-48.60	[-49.39; -47.81]	47.3%	4.2%
Takeuchi H et.al. 2010	32	-33.10	5.1098	-33.10	[-34.87; -31.33]	9.5%	4.2%
Byerly MJ et.al. 2009	105	-34.09	8.0410	-34.09	[-35.63; -32.56]	12.5%	4.2%
Woo Y.S. et al. 2016	77	-28.00	65.3445	-28.00	[-42.60; -13.40]	0.1%	4.0%
Kelly DL et.al. 2021	50	-29.38	19.7198	-29.38	[-34.85; -23.91]	1.0%	4.2%
Woo Y.S. et.al. 2019	33	-16.80	87.1090	-16.80	[-46.52; 12.92]	0.0%	3.4%
Ichinose M et.al. 2021	27	-15.90	23.3245	-15.90	[-24.70; -7.10]	0.4%	4.1%
Kinon, BJ et al. 2000	45	-32.26	33.0670	-32.26	[-41.92; -22.60]	0.3%	4.1%
Montejo A.L. et al. 2009	20	-47.48	46.3029	-47.48	[-67.77; -27.19]	0.1%	3.8%
Huang P et.al. 2011	34	-95.68	36.8447	-95.68	[-108.06; -83.30]	0.2%	4.0%
Jen Y.W. et.al. 2020	63	-47.00	58.5982	-47.00	[-61.47; -32.53]	0.1%	4.0%
Takeuchi H et al. 2008	53	-36.40	4.5033	-36.40	[-37.61; -35.19]	20.2%	4.2%
Hwang TJ et.al. 2015	79	-45.43	10.7946	-45.43	[-47.81; -43.05]	5.2%	4.2%
Kim SW et.al. 2009	61	-40.70	41.2466	-40.70	[-51.05; -30.35]	0.3%	4.1%
Nishimoto M et.al. 2012	7	-88.80	81.4654	-88.80	[-149.15; -28.45]	0.0%	2.1%
Hashimoto N et.al. 2015	22	-13.20	27.1448	-13.20	[-24.54; -1.86]	0.2%	4.1%
**Common effect model**	**1273**			**-42.55**	**[-43.09; -42.01]**	**100.0%**	**--**
**Random effects model**				**-49.30**	**[-61.41; -37.19]**	**--**	**100.0%**

Heterogeneity: I^2 = 98%, τ^2 = 907.1595, p < 0.01

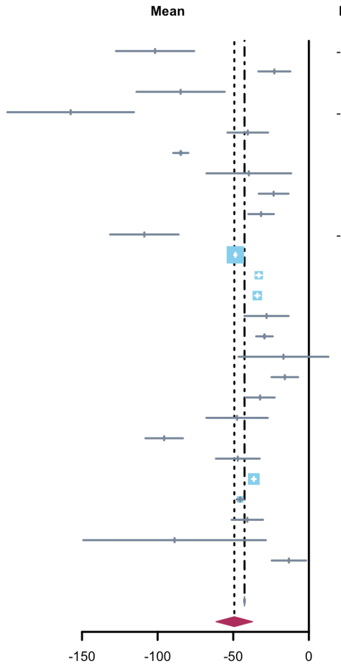

Supplementary Figure 2. Forest plot of single-arm meta-analysis on switching to another antipsychotic.

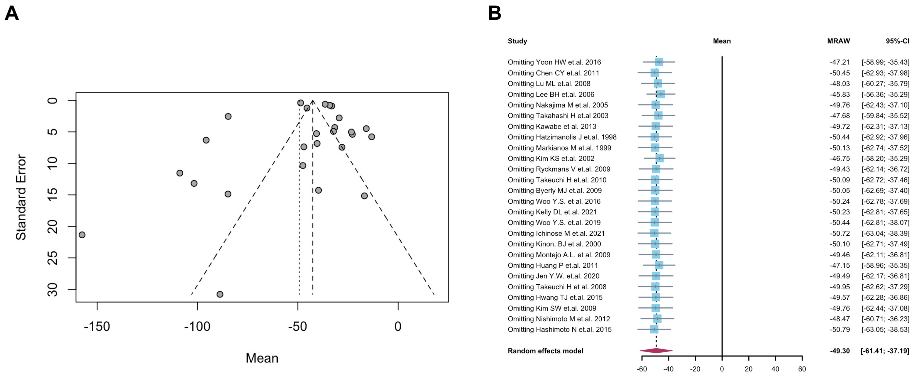

Supplementary Figure 3. A, Funnel plot of single-arm meta-analysis on switching to another antipsychotic; B, Sensitive analysis.

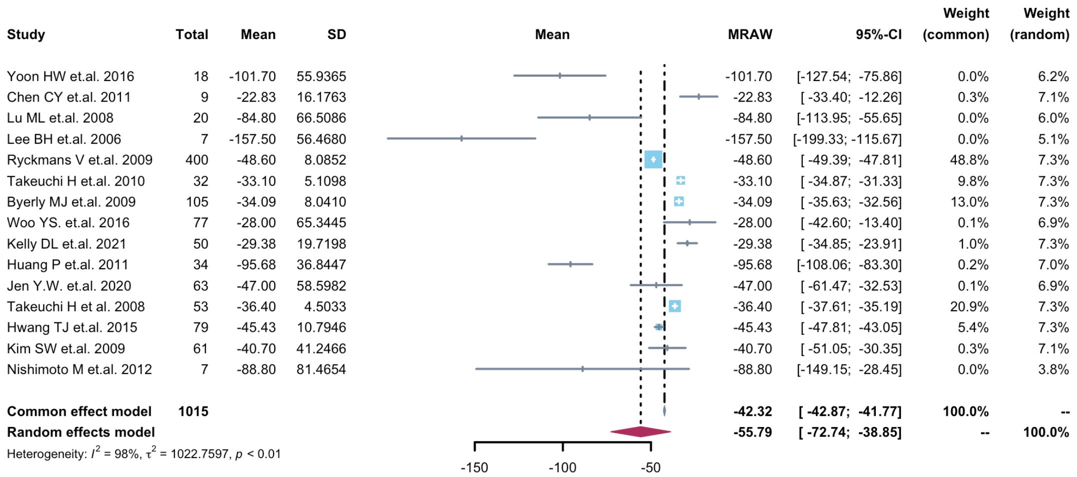

Supplementary Figure 4. Forest plot of single-arm meta-analysis on switching to aripiprazole.

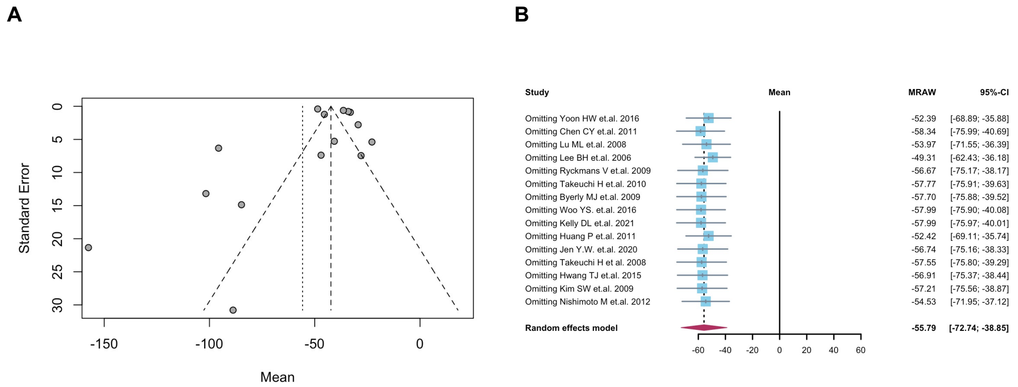

Supplementary Figure 5. A, Funnel plot of single-arm meta-analysis on switching to aripiprazole; B, Sensitive analysis.

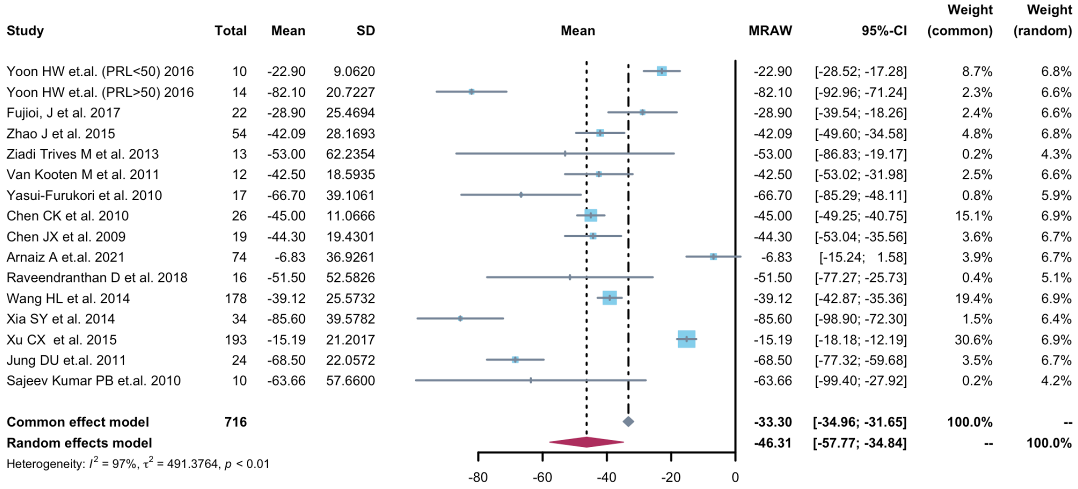

Supplementary Figure 6. Forest plot of single-arm meta-analysis on adjunctive aripiprazole.

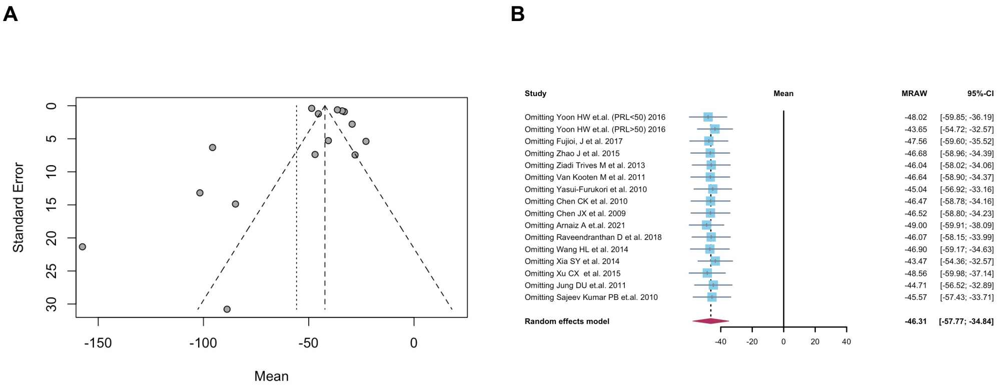

Supplementary Figure 7. A, Funnel plot of single-arm meta-analysis on adjunctive aripiprazole; B, Sensitive analysis.

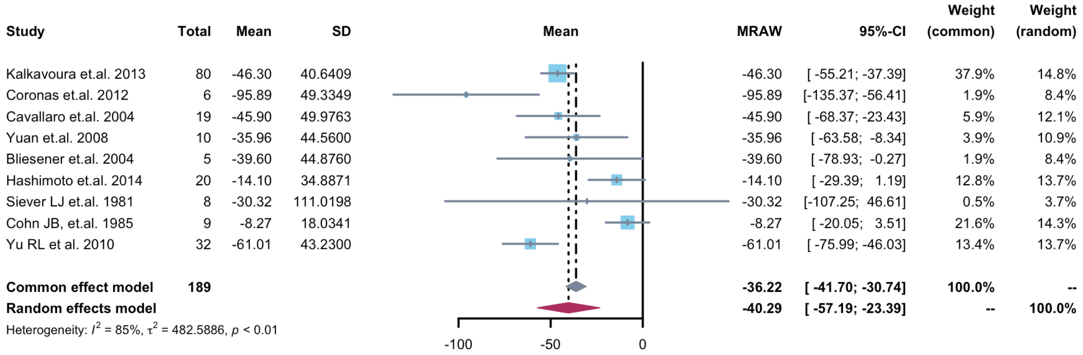

Supplementary Figure 8. Forest plot of single-arm meta-analysis on adjunctive dopamine agonist.

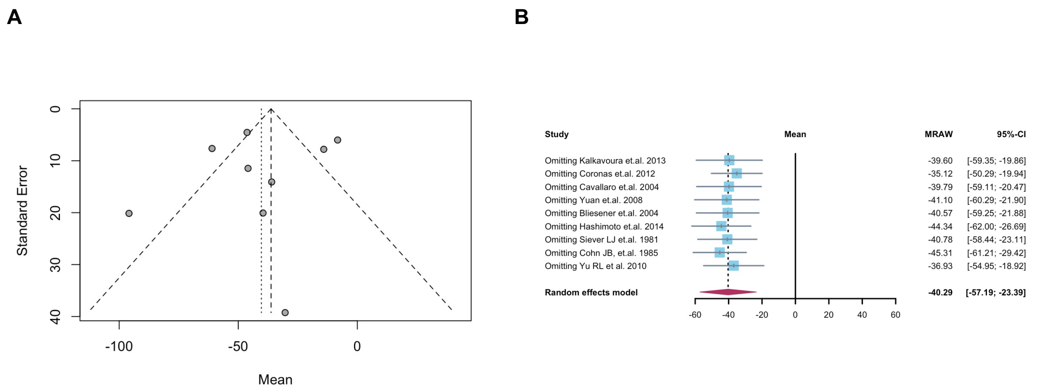

Supplementary Figure 9. A, Funnel plot of single-arm meta-analysis on adjunctive dopamine agonist; B, Sensitive analysis.

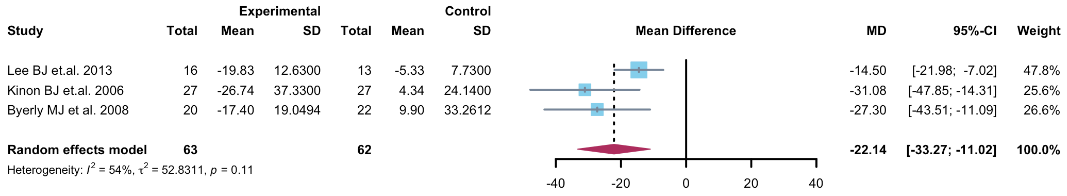

Supplementary Figure 10. Forest plot of RCT meta-analysis on switching to another antipsychotic.

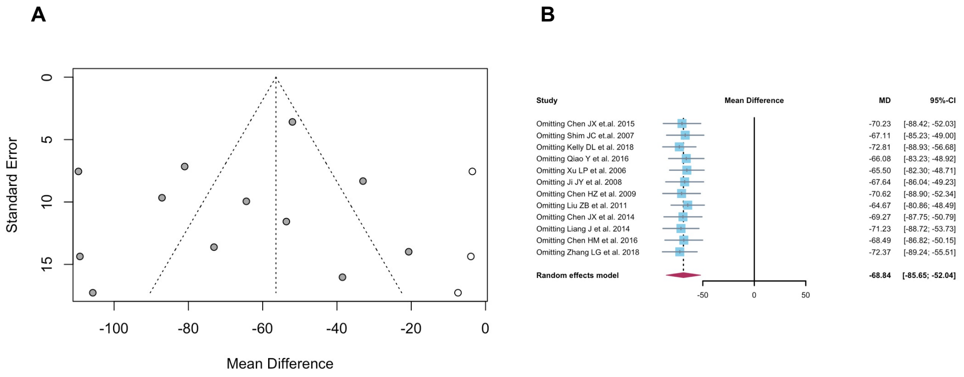

Supplementary Figure 11. A, Funnel plot of RCT meta-analysis on adjunctive aripiprazole; B, Sensitive analysis.

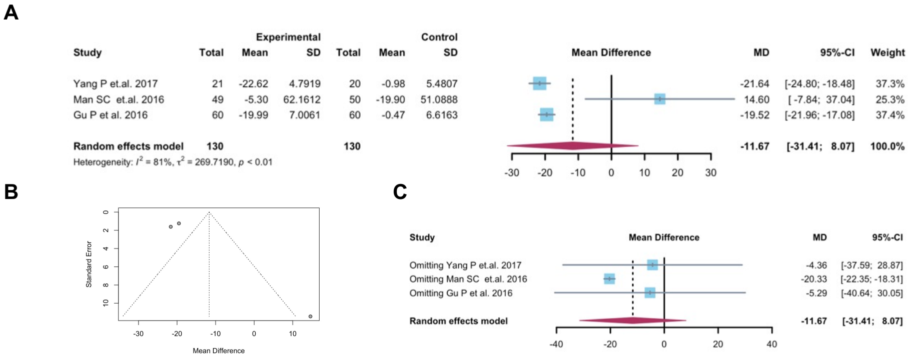

Supplementary Figure 12. A, forest plot of RCT meta-analysis on adjunctive Peony-Glycyrrhiza decoction; B, Funnel plot of RCT meta-analysis on adjunctive aripiprazole; C, Sensitive analysis.

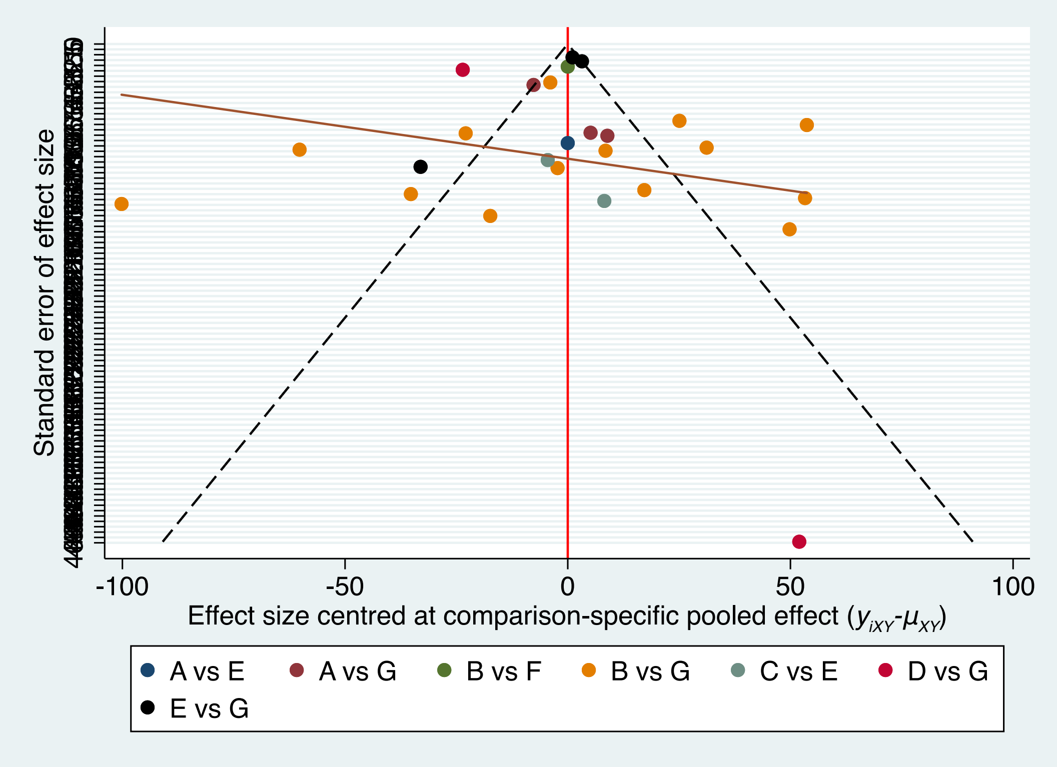

Supplementary Figure 13. Funnel plot of network meta-analysis: all strategies. A, switching to another antipsychotic; B, adjunctive aripiprazole; C, adjunctive dopamine agonist; D, adjunctive metformin; E, adjunctive Peony-Glycyrrhiza decoction; F, placebo.

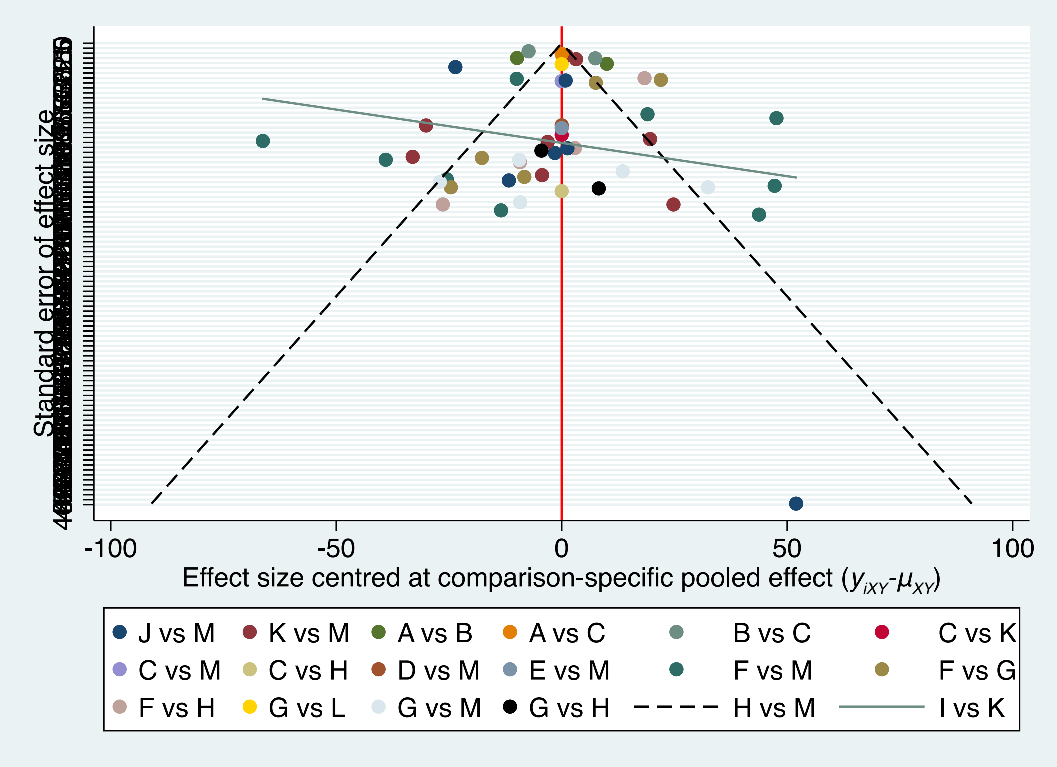

Supplementary Figure 14. Funnel plot of network meta-analysis: based on different dosage and switching options.

A, switching to ARI with fixed dosage and reducing the previous antipsychotic immediately; B, switching to ARI with fixed dosage and reducing the previous antipsychotic in tardation; C, switching to ARI in titration and reducing the previous antipsychotic in tardation; D, switching to quetiapine; E, switching to olanzapine; F, adjunctive 5mg aripiprazole; G, adjunctive 10mg aripiprazole; H, adjunctive more than 10mg aripiprazole; I, adjunctive dopamine agonist; J, adjunctive metformin; K, adjunctive Peony-Glycyrrhiza decoction; L, adjunctive high-dose vitamin B6; M, placebo.

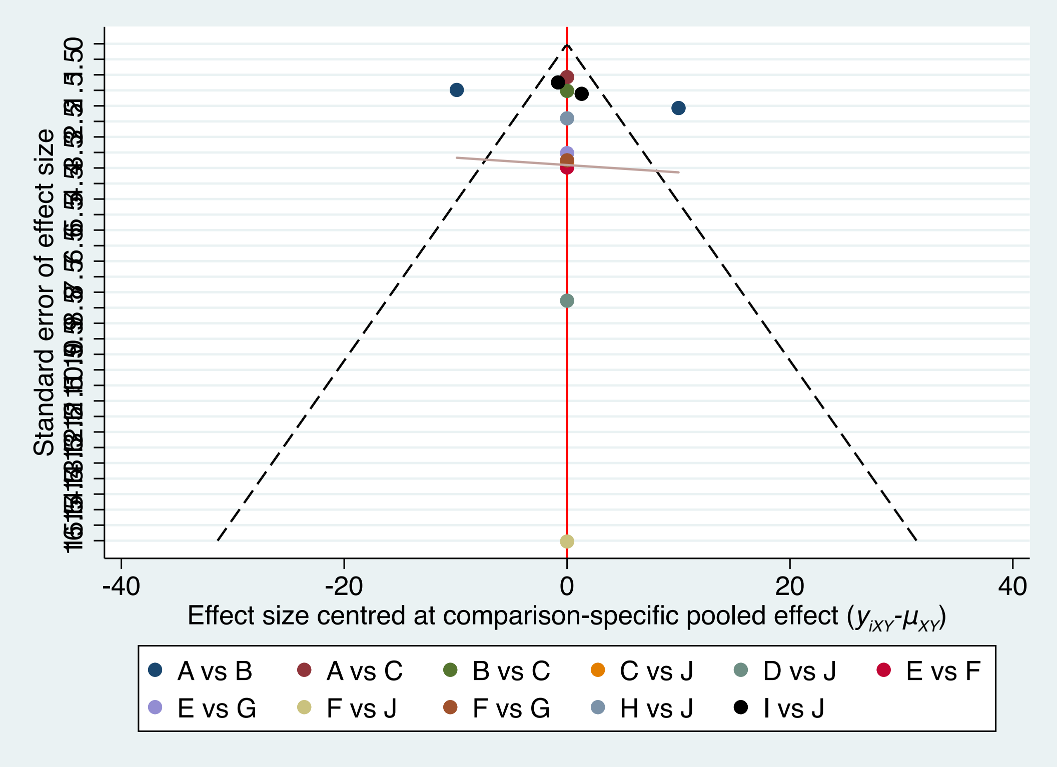

Supplementary Figure 15. Funnel plot of network meta-analysis: baseline prolactin < 50 ng/ml.

A, switching to ARI with fixed dosage and reducing the previous antipsychotic immediately; B, switching to ARI with fixed dosage and reducing the previous antipsychotic in tardation; C, switching to ARI in titration and reducing the previous antipsychotic in tardation; D, switching to quetiapine; E, adjunctive 5mg aripiprazole; F, adjunctive 10mg aripiprazole; G, adjunctive more than 10mg aripiprazole; H, adjunctive dopamine agonist; I, adjunctive Peony-Glycyrrhiza decoction; J, placebo.

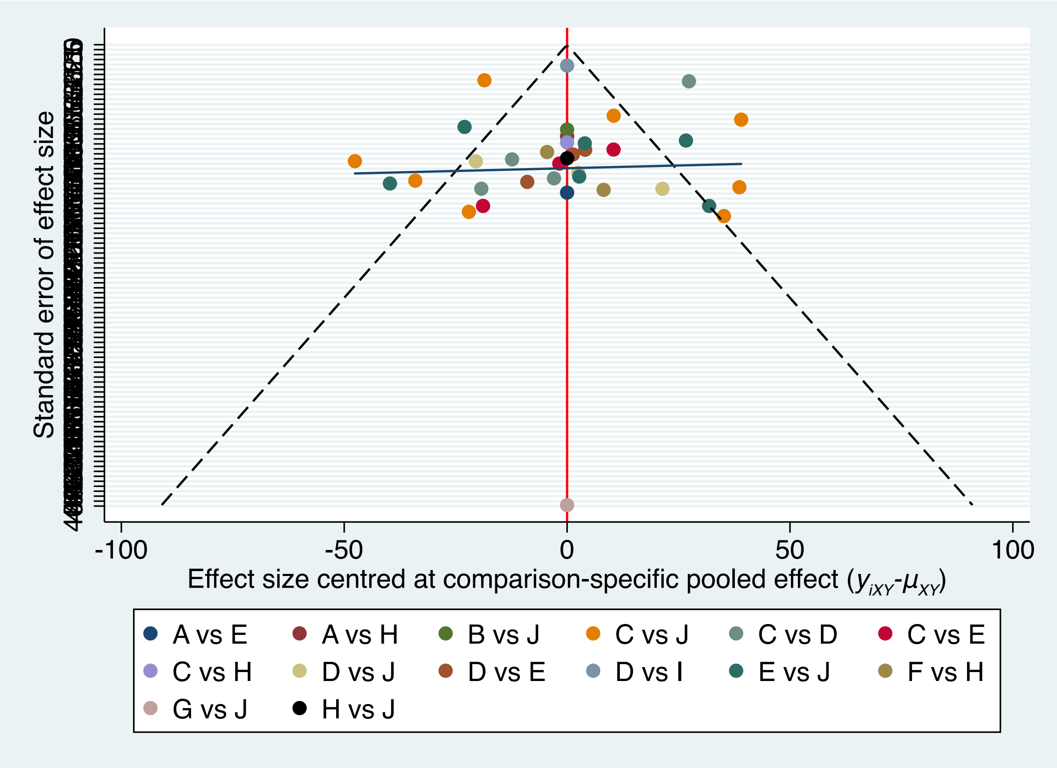

Supplementary Figure 16. Funnel plot of network meta-analysis: baseline prolactin > 50 ng/ml.

A, switching to ARI in titration and reducing the previous antipsychotic in tardation; B, switching to olanzapine; C, adjunctive 5mg aripiprazole; D, adjunctive 10mg aripiprazole; E, adjunctive more than 10mg aripiprazole; F, adjunctive dopamine agonist; G, adjunctive metformin; H, adjunctive Peony-Glycyrrhiza decoction; I, adjunctive high-dose vitamin B6; J, placebo.

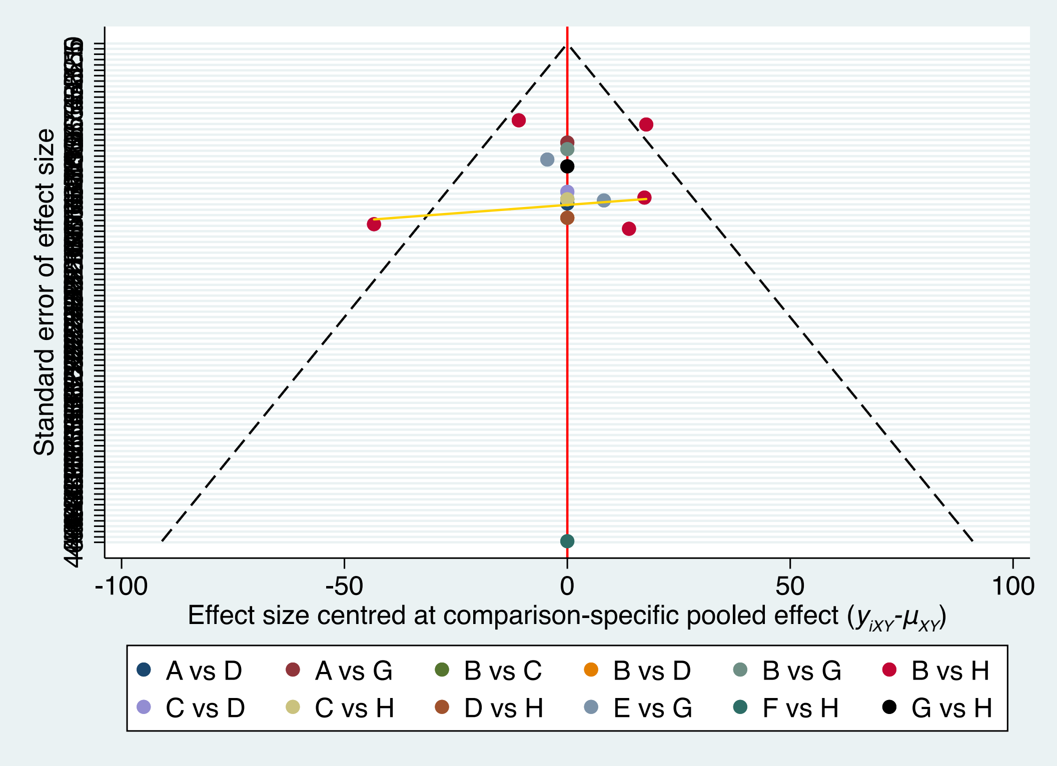

Supplementary Figure 17. Funnel plot of network meta-analysis: baseline prolactin > 100 ng/ml.
A, switching to ARI in titration and reducing the previous antipsychotic in tardation; B, adjunctive 5mg aripiprazole; C, adjunctive 10mg aripiprazole; D, adjunctive more than 10mg aripiprazole; E, adjunctive dopamine agonist; F, adjunctive metformin; G, adjunctive Peony-Glycyrrhiza decoction; H, placebo.

Supplementary Results

In the subgroup of 50 to 100 ng/ml, 5 strategies including adjunctive 5mg ARI, adjunctive 10mg ARI, adjunctive more than 10mg ARI, switching to OLA and adjunctive vitamin B6 were included (9 studies with 881 participants). When compare to the placebo, adjunctive ARI (5mg: MD = -43.84, 95% CI = -66.85 to -18.27; 10mg: MD = -43.90, 95% CI = -68.51 to -19.22; more than 10mg: MD = -61.42, 95% CI = -84.81 to -38.20) and vitamin B6 (MD = -75.95, 95% CI = -125.74 to -26.13) showed the significant effect of decreasing PRL level, while the difference of PRL reduction between switching to OLA and placebo was not significant.

Supplementary Tables

Supplementary Table 1. Summary of findings tables

Summary of findings tables			
Outcomes	Intervention	MD (95% CI) Network estimate	Certainty of evidence
Reduction of prolactin level from baseline	ARI<br>(15 RCTs, 694 participants)	-60.21 (-78.36, -41.89)	⊕⊕⊕⊕ High
	VitB6<br>(1 RCT, 100 participants)	-91.98 (-159.55, -25.78)	⊕⊕⊕○ Moderate Due to indirectness
	Switching<br>(5 RCTs, 115 participants)	-38.23 (-68.76, -8.04)	⊕⊕⊕○ Moderate Due to risk of bias
	MET<br>(2 RCTs, 114 participants)	-18.49 (-75.91, 34.31)	⊕⊕○○ Low Due to severe impercision
	PGD<br>(7 RCTs, 236 participants)	-26.77 (-56.82, 3.17)	⊕⊕○○ Low Due to heterogeneity and impercision
	DA<br>(2 RCTs, 42 participants)	-31.69 (-89.00, 25.92)	⊕○○○ Very low Due to indirectness and severe impercision
	Placebo	Reference comparator	Reference comparator
Reduction of prolactin level from baseline (stratified by the	ARI_more_10mg<br>(8 RTCs, 346 participants)	-62.07 (-84.71, -39.59)	⊕⊕⊕⊕ High
	ARI_5mg<br>(11 RTCs, 486 participants)	-61.21 (-80.23, -42.33)	⊕⊕⊕⊕ High

dosage of ARI and different Switching strategies)	ARI_10mg<br>(7 RTCs, 233 participants)	-55.97 (-78.66, -33.14)	⊕⊕⊕⊕ High
	Switch_ARI_ti_ta<br>(5 RTCs, 303 participants)	-44.53 (-81.76, -7.98)	⊕⊕⊕○ Moderate Due to risk of bias
	PGD<br>(7 RCTs, 236 participants)	-27.84 (-55.23, -0.29)	⊕⊕⊕○ Moderate Due to heterogeneity
	Switch_OLA<br>(1 RCT, 27 participants)	-31.11 (-90.13, 27.60)	⊕⊕○○ Low Due to severe impercision
	Switch_QUE<br>(1 RCT, 20 participants)	-27.24 (-86.89, 32.31)	⊕⊕○○ Low Due to severe impercision
	MET<br>(2 RCTs, 114 participants)	-16.62 (-68.67, 31.29)	⊕⊕○○ Low Due to severe impercision
	VitB6<br>(1 RCT, 100 participants)	-87.35 (-148.31, -26.15)	⊕⊕⊕○ Moderate Due to indirectness
	Switch_ARI_fixed_im<br>(2 RCTs, 76 participants)	-52.74 (-112.00, 5.10)	⊕⊕○○ Low Due to indirectness and impercision
	Switch_ARI_fixed_ta<br>(3 RCTs, 273 participants)	-49.80 (-103.02, 2.45)	⊕⊕○○ Low Due to indirectness and impercision
	DA<br>(2 RCTs, 42 participants)	-32.73 (-83.62, 19.00)	⊕○○○ Very low Due to indirectness and severe impercision
Reduction of prolactin level from baseline	ARI_more _10mg<br>(1 RCT, 64 participants)	-37.80 (-101.65, 27.22)	⊕⊕⊕○ Moderate Due to impercision
	PGD<br>(2 RCTs, 81 participants)	-20.53 (-48.77, 8.11)	⊕⊕⊕○ Moderate Due to impercision

(baseline PRL < 50 ng/ml)	ARI_5mg<br>(1 RCT, 65 participants)	-38.94 (-103.39, 25.34)	⊕⊕○○ Low Due to indirectness and impercision
	ARI_10mg<br>(2 RCTs, 84 participants)	-38.30 (-88.17, 11.17)	⊕⊕○○ Low Due to indirectness and impercision
	Switch_QUE<br>(1 RCT, 20 participants)	-26.92 (-70.03, 15.57)	⊕⊕○○ Low Due to severe impercision
	Switch_ARI_ti_ta<br>(2 RCTs, 51 participants)	-14.42 (-56.87, 27.00)	⊕⊕○○ Low Due to severe impercision
	MET<br>(1 RCT, 72 participants)	-1.10 (-42.33, 39.15)	⊕⊕○○ Low Due to severe impercision
	Switch_ARI_fixed_im<br>(2 RCTs, 76 participants)	-25.53 (-83.83, 30.66)	⊕○○○ Very low Due to indirectness and severe impercision
	Switch_ARI_fixed_ta<br>(2 RCTs, 73 participants)	-24.03 (-81.72, 32.19)	⊕○○○ Very low Due to indirectness and severe impercision
Reduction of prolactin level from baseline (50 < baseline PRL < 100 ng/ml)	ARI_10mg<br>(5 RCTs, 247 participants)	-61.42 (-84.81, -38.20)	⊕⊕⊕⊕ High
	ARI_more_10mg<br>(4 RCTs, 130 participants)	-43.90 (-68.51, -19.22)	⊕⊕⊕⊕ High
	ARI_5mg<br>(4 RCTs, 171 participants)	-43.84 (-66.85, -18.27)	⊕⊕⊕⊕ High
	VitB6<br>(1 RCT, 100 participants)	-75.95 (-125.74, -26.13)	⊕⊕⊕○ Moderate Due to indirectness
	Switch_OLA<br>(1 RCT, 27 participants)	-31.02 (-76.76, 15.06)	⊕⊕○○ Low Due to severe impercision

Reduction of prolactin level from baseline (baseline PRL > 100 ng/ml)	ARI_5mg<br>(6 RCTs, 250 participants)	-81.251 (-126.00, -35.45)	⊕⊕⊕⊕ High
	ARI_more_10mg<br>(2 RCTs, 39 participants)	-79.72 (-165.17, 4.95)	⊕⊕⊕○ Moderate Due to impercision
	ARI_10mg<br>(1 RCT, 15 participants)	-85.95 (-179.85, 8.92)	⊕⊕○○ Low Due to severe impercision
	MET<br>(1 RCT, 42 participants)	-75.88 (-212.69, 62.80)	⊕⊕○○ Low Due to severe impercision
	PGD<br>(5 RCTs, 155 participants)	-54.19 (-128.02, 19.11)	⊕⊕○○ Low Due to heterogeneity and impercision
	Switch_ARI_ti_ta<br>(2 RCTs, 52 participants)	-86.63 (-186.71, 12.77)	⊕○○○ Very low Due to risk of bias, indirectness and impercision
	DA<br>(2 RCTs, 42 participants)	-58.43 (-163.32, 47.24)	⊕○○○ Very low Due to indirectness and severe impercision
Reduction of prolactin level from baseline (baseline PRL > 50 ng/ml)	ARI_more_10 mg<br>(6 RCTs, 262 participants)	-68.01 (-97.12, -39.72)	⊕⊕⊕⊕ High
	ARI_5mg<br>(10 RCTs, 421 participants)	-64.26 (-87.00, -41.37)	⊕⊕⊕⊕ High
	ARI_10mg<br>(6 RCTs, 169 participants)	-59.81 (-90.10, -29.76)	⊕⊕⊕⊕ High
	VitB6<br>(1 RCT, 100 participants)	-91.84 (-165.31, -17.74)	⊕⊕⊕○ Moderate Due to indirectness
	MET<br>(1 RCT, 42 participants)	-76.20 (-191.38, 37.08)	⊕⊕⊕○ Moderate Due to impercision

	Switch_ARI_ti_ta (2 RCTs, 52 participants)	-74.80 (-134.22, -15.99)	⊕⊕○○ Low Due to risk of bias and indirectness
	DA (2 RCTs, 42 participants)	-49.56 (-119.15, 18.69)	⊕⊕○○ Low Due to indirectness and impercision
	PGD (5 RCTs, 155 participants)	-44.87 (-91.93, 2.17)	⊕⊕○○ Low Due to heterogeneity and impercision
	Switch_OLA (1 RCT, 27 participants)	-30.78 (-100.59, 39.34)	⊕⊕○○ Low Due to severe impercision

ARI_5mg, adjunctive 5mg aripiprazole; ARI_10mg, adjunctive 10mg aripiprazole; ARI_more_10mg, adjunctive more than 10mg aripiprazole; DA, adjunctive dopamine agonist; MET, adjunctive metformin; PGD, adjunctive Peony-Glycyrrhiza decoction; switch_ARI_fixed_im, switching to ARI with fixed dosage and reducing the previous antipsychotic immediately; switch_ARI_fixed_ta, switching to ARI with fixed dosage and reducing the previous antipsychotic in tardation; switch_ARI_ti_ta, switching to ARI in titration and reducing the previous antipsychotic in tardation; switch_OLA, switching to olanzapine; switch_QUE, switching to quetiapine; VitB6, adjunctive high-dose vitamin B6.

Supplementary Table 2. Minimally contextualized framework

All strategies			
High certainly	Intervention	MD (95% CI)	Surface under the cumulative ranking curve
category 2: among the most effective			
category 1: inferior to the most effective, or superior to the least effective	ARI	-60.21 (-78.36, -41.89)	0.80
	Switching	-38.23 (-68.76, -8.04)	0.54
category 0: among the least effective	PGD	-26.77 (-56.82, 3.17)	0.38
	MET	-18.49 (-75.91, 34.31)	0.32
Low certainly	Intervention	MD (95% CI)	Surface under the cumulative ranking curve
category 2: among the most effective			
category 1: inferior to the most effective, or superior to the least effective	VitB6	-91.98 (-159.55, -25.78)	0.93
category 0: among the least effective	DA	-31.69 (-89.00, 25.92)	0.45
All strategies divided based on dosage			
High certainly	Intervention	MD (95% CI)	Surface under the cumulative ranking curve
category 2: among the most effective	ARI_more_10mg	-62.07 (-84.71, -39.59)	0.75
	ARI_5mg	-61.21 (-80.23, -42.33)	0.74
category 1: inferior to the most effective, or superior to the least effective	ARI_10mg	-55.97 (-78.66, -33.14)	0.66
	Switch_ARI_ti_ta	-44.53 (-81.76, -7.98)	0.51

	PGD	-27.84 (-55.23, -0.29)	0.31
category 0: among the least effective	Switch_OLA	-31.11 (-90.13, 27.60)	0.39
	Switch_QUE	-27.24 (-86.89, 32.31)	0.35
	MET	-16.62 (-68.67, 31.29)	0.25
Low certainly	Intervention	MD (95% CI)	Surface under the cumulative ranking curve
category 2: among the most effective			
category 1: inferior to the most effective, or superior to the least effective	VitB6	-87.35 (-148.31, -26.15)	0.89
category 0: among the least effective	Switch_ARI_fixed_im	-52.74 (-112.00, 5.10)	0.61
	Switch_ARI_fixed_ta	-49.80 (-103.02, 2.45)	0.58
	DA	-32.73 (-83.62, 19.00)	0.39
PRL < 50 ng/ml			
High certainly	Intervention	MD (95% CI)	Surface under the cumulative ranking curve
category 2: among the most effective			
category 1: inferior to the most effective, or superior to the least effective			
category 0: among the least effective	ARI_10mg	-38.30 (-88.17, 11.17)	0.71
	PGD	-20.53 (-48.77, 8.11)	0.49
	Switch_QUE	-26.92 (-70.03, 15.57)	0.59
	Switch_ARI_ti_ta	-14.42 (-56.87, 27.00)	0.37
	MET	-1.10 (-42.33, 39.15)	0.20

Low certainly	Intervention	MD (95% CI)	Surface under the cumulative ranking curve
category 2: among the most effective			
category 1: inferior to the most effective, or superior to the least effective			
category 0: among the least effective	ARI_5mg	-38.94 (-103.39, 25.34)	0.70
	ARI_more_10mg	-37.80 (-101.65, 27.22)	0.68
	Switch_ARI_fixed_im	-25.53 (-83.83, 30.66)	0.57
	Switch_ARI_fixed_ta	-24.03 (-81.72, 32.19)	0.54
50 ng/ml < PRL < 100 ng/ml			
High certainly	Intervention	MD (95% CI)	Surface under the cumulative ranking curve
category 2: among the most effective			
category 1: inferior to the most effective, or superior to the least effective	ARI_10mg	-61.42 (-84.81, -38.20)	0.80
	ARI_more_10mg	-43.90 (-68.51, -19.22)	0.48
	ARI_5mg	-43.84 (-66.85, -18.27)	0.47
category 0: among the least effective	Switch_OLA	-31.02 (-76.76, 15.06)	0.34
Low certainly	Intervention	MD (95% CI)	Surface under the cumulative ranking curve
category 2: among the most effective			
category 1: inferior to the most effective, or superior to the least effective	VitB6	-75.95 (-125.74, -26.13)	0.90
PRL > 100 ng/ml			

High certainly	Intervention	MD (95% CI)	Surface under the cumulative ranking curve
category 2: among the most effective			
category 1: inferior to the most effective, or superior to the least effective	ARI_5mg	-81.251 (-126.00, -35.45)	0.63
category 0: among the least effective	ARI_10mg	-85.95 (-179.85, 8.92)	0.65
	ARI_more_10mg	-79.72 (-165.17, 4.95)	0.61
	MET	-75.88 (-212.69, 62.80)	0.56
	PGD	-54.19 (-128.02, 19.11)	0.39
Low certainly	Intervention	MD (95% CI)	Surface under the cumulative ranking curve
category 2: among the most effective			
category 1: inferior to the most effective, or superior to the least effective			
category 0: among the least effective	Switch_ARI_ti_ta	-86.63 (-186.71, 12.77)	0.66
	DA	-58.43 (-163.32, 47.24)	0.45
PRL > 50 ng/ml			
High certainly	Intervention	MD (95% CI)	Surface under the cumulative ranking curve
category 2: among the most effective			
category 1: inferior to the most effective, or superior to the least effective	ARI_more_10mg	-68.01 (-97.12, -39.72)	0.64
	ARI_5mg	-64.26 (-87.00, -41.37)	0.59
	ARI_10mg	-59.81 (-90.10, -29.76)	0.52

category 0: among the least effective	MET	-76.20 (-191.38, 37.08)	0.63
	PGD	-44.87 (-91.93, 2.17)	0.36
	Switch_OLA	-30.78 (-100.59, 39.34)	0.28
Low certainly	Intervention	MD (95% CI)	Surface under the cumulative ranking curve
category 2: among the most effective			
category 1: inferior to the most effective, or superior to the least effective	VitB6	-91.84 (-165.31, -17.74)	0.79
	Switch_ARI_ti_ta	-74.80 (-134.22, -15.99)	0.70
category 0: among the least effective	DA	-49.56 (-119.15, 18.69)	0.44

PRL, prolactin; ARI_5mg, adjunctive 5mg aripiprazole; ARI_10mg, adjunctive 10mg aripiprazole; ARI_more_10mg, adjunctive more than 10mg aripiprazole; DA, adjunctive dopamine agonist; MET, adjunctive metformin; PGD, adjunctive Peony-Glycyrrhiza decoction; switch_ARI_fixed_im, switching to ARI with fixed dosage and reducing the previous antipsychotic immediately; switch_ARI_fixed_ta, switching to ARI with fixed dosage and reducing the previous antipsychotic in tardation; switch_ARI_ti_ta, switching to ARI in titration and reducing the previous antipsychotic in tardation; switch_OLA, switching to olanzapine; switch_QUE, switching to quetiapine; VitB6, adjunctive high-dose vitamin B6.

Supplementary Table 3. Risk of bias: RCT (ROB 2).

study	Randomization process	Deviation from intended interventions	Missing outcome	Measurement of the outcome	Selection of the reported result	over all Bias	weight
Byerly MJ et al., 2008	Some concerns	Low	Low	Low	Low	Some concerns	42
Byerly MJ et al., 2009	Some concerns	Low	Low	Low	Low	Some concerns	105
Chen HM et al., 2016	Low	Low	Low	Low	Low	Low	61
Chen HZ et al., 2009	Low	Low	Low	Low	Low	Low	65
Chen JX et.al., 2014	Some concerns	Low	Low	Low	Low	Some concerns	116
Chen JX et.al., 2015	Some concerns	Low	Low	Low	Low	Some concerns	120
Gu P et.al., 2016	Low	Low	Low	Low	Low	Low	120
Huang P et.al., 2011	Some concerns	Low	Low	Low	Low	Some concerns	66
Hwang TJ et.al., 2015	Low	Low	Low	Low	Low	Low	79
Ji JY et.al., 2008	Low	Low	Low	Low	Low	Low	117
Kelly DL et.al., 2018	Some concerns	Low	Low	Low	Low	Some concerns	42
Kinon BJ et.al, 2006	Some concerns	Low	Low	Low	Low	Some concerns	54
Lee BJ et.al., 2013	Some concerns	Low	Low	Low	Low	Some concerns	29
Liang J et.al., 2014	Low	Low	Low	Low	Low	Low	40
Liu ZB et.al., 2011	Some concerns	Low	Low	Some concerns	Some concerns	Some concerns	142
Man SC et.al., 2016	Low	Low	Low	Low	Low	Low	99
Qiao Y et.al., 2016	Some concerns	Low	Low	Low	Low	Some concerns	60
Ryckmans V et.al., 2009	Some concerns	Low	Low	Low	Low	Some concerns	400
Shim JC et.al., 2007	Some concerns	Low	Low	Low	Low	Some concerns	54
Wang HL et.al., 2014	Low	Low	Low	Low	Low	Low	178
Wu RR et.al., 2012	Low	Low	Low	Low	Low	Low	84

Xia JX et.al., 2011	Some concerns	Low	Low	Low	Low	Some concerns	143
Xia SY et.al., 2014	Low	Low	Low	Low	Low	Low	67
Xu CX et.al., 2015	Low	Low	Low	Low	Low	Low	193
Xu LP et.al., 2006	Some concerns	Low	Low	Low	Low	Some concerns	60
Yang P et.al., 2017	Some concerns	Low	Low	Low	Low	Some concerns	41
Yoon HW et.al., 2016	Some concerns	Low	Low	Low	Low	Some concerns	42
Yu RL et.al., 2010	Some concerns	Low	Low	Some concerns	Low	Some concerns	63
Yuan et.al., 2008	Some concerns	Low	Low	Low	Low	Some concerns	20
Zhang LG et.al., 2018	Low	Low	Low	Low	Low	Low	58
Zhuo CJ et.al., 2021	Low	Low	Low	Low	Low	Low	194

RoB 2, Cochrane Risk of Bias tool 2.

Supplementary Table 4. Risk of bias: non-RCT (MINORS).

study	A clearly stated aim	Inclusion of consecutive patients	Prospective collection of data	Endpoint appropriate for aim of study	Unbiased assessment of study endpoint	Follow-up period appropriate	Loss of follow-up less than 5%	Prospective calculation of study size
Yoon HW et.al. 2016	2	2	2	2	2	1	0	0
Chen CY et.al. 2011	2	1	2	2	2	1	2	0
Lu ML et.al. 2008	2	2	2	2	2	1	2	0
Lee BH et.al. 2006	2	2	2	2	2	1	0	0
Nakajima M et.al. 2005	2	1	2	2	2	1	0	0
Takahashi H et.al 2003	2	0	2	2	2	1	1	0
Kawabe et al. 2013	2	0	2	2	2	1	2	0
Hatzimanolis J et.al. 1998	2	2	2	2	2	1	1	0
Markianos M et.al. 1999	2	2	2	2	2	1	1	0

Kim KS et.al. 2002	2	2	2	2	2	1	0	0
Ryckmans V et.al. 2009	2	2	2	2	2	1	2	2
Takeuchi H et.al. 2010	2	1	2	2	2	1	1	0
Byerly MJ et.al. 2009	2	1	2	2	2	1	0	0
Woo Y.S. et al. 2016	2	1	2	2	2	1	2	0
Kelly DL et.al. 2021	2	1	2	2	2	1	2	0
Woo Y.S. et.al. 2019	2	2	2	2	2	1	1	0
Ichinose M et.al. 2021	2	1	2	2	2	1	0	0
Kinon, BJ et al. 2000	2	1	2	2	2	1	0	0
Montejo A.L. et al. 2009	2	1	2	2	2	1	2	0
Huang P et.al. 2011	2	2	2	2	2	1	2	0
Jen Y.W. et.al. 2020	2	2	2	2	2	1	0	0
Takeuchi H et al. 2008	2	2	2	2	2	1	2	0
Hwang TJ et.al. 2015	2	2	1	2	2	1	2	0
Kim SW et.al. 2009	2	1	2	2	2	1	2	0
Nishimoto M et.al. 2012	2	1	2	2	2	1	0	0
Hashimoto N et.al. 2015	2	1	2	2	2	1	1	0
Fujioi J et al. 2017	2	2	2	2	2	1	2	0
Zhao J et al. 2015	2	2	2	2	2	1	2	0
Ziadi Trives M et al. 2013	2	1	2	2	2	1	0	0
Van Kooten M et al. 2011	2	2	2	2	2	1	0	0
Yasui-Furukori et al. 2010	2	1	2	2	2	1	2	0
Chen CK et al. 2010	2	2	2	2	2	1	2	0
Chen JX et al. 2009	2	2	2	2	2	1	2	0
Arnaiz A et.al. 2021	2	0	2	2	2	1	1	0

Raveendranthan D et al. 2018	2	0	2	2	2	1	1	0
Wang HL et al. 2014	2	2	2	2	2	1	2	0
Xia SY et al. 2014	2	2	2	2	2	1	2	0
Xu CX et al. 2015	2	2	2	2	2	1	2	0
Jung DU et.al. 2011	2	1	2	2	2	1	1	0
Sajeev Kumar PB et.al. 2010	2	0	2	2	2	1	2	0
Kalkavoura et.al. 2013	2	2	2	2	2	1	2	0
Coronas et.al. 2012	2	2	2	2	2	1	2	0
Cavallaro et.al. 2004	2	1	2	2	2	1	2	0
Yuan et.al. 2008	2	2	2	2	2	1	2	0
Bliesener et.al. 2004	2	0	2	2	2	1	2	0
Hashimoto et.al. 2014	2	2	2	2	2	1	2	0
Siever LJ et.al. 1981	2	1	2	2	2	1	2	0
Cohn JB et.al. 1985	2	0	2	2	2	1	2	0
Yu RL et al. 2010	2	2	2	2	2	1	2	0

MINORS, Methodological Index for Non-Randomized Studies.

0, not report; 1, report but inadequate; 2, Report and adequate.
